# Recognition and linking of discontinuous named entities in healthcare: a comparative performance analysis

**DOI:** 10.3389/fdgth.2026.1758921

**Published:** 2026-06-09

**Authors:** Areej Alhassan, Viktor Schlegel, Rina Carines Cabral, Riza Batista-Navarro, Soyeon Caren Han, Josiah Poon, Goran Nenadic

**Affiliations:** 1 School of Computer Science, The University of Manchester, Manchester, United Kingdom; 2 Department of Management Information Systems, King Saud University, Riyadh, Saudi Arabia; 3 Imperial Global Singapore, Imperial College London, Singapore, Singapore; 4 Department of Bioengineering, Imperial College London, London, United Kingdom; 5 School of Computer Science, The University of Sydney, Sydney, NSW, Australia; 6 School of Computing and Information Systems, The University of Melbourne, Melbourne, VIC, Australia

**Keywords:** clinical named entity recognition, discontinuous named entity recognition, healthcare NLP, human phenotype extraction, named entity normalisation, natural language processing, occupational substance exposure

## Abstract

**Introduction:**

The recognition and linking of discontinuous named entities (DiscNEs) in healthcare remain challenging due to their fragmented structure and semantic complexity. This study presents a comparative analysis of two state-of-the-art DiscNER models: TriG-NER, a grid-tagging architecture, and DocDiscNER, a generative document-level model. The aim is to provide a broader understanding of their generalisation capabilities, performance across diverse entity categories, and effectiveness when integrated with a Named Entity Normalisation (NEN) component.

**Methods:**

Experiments were conducted on two healthcare corpora with distinct characteristics: BioCreative-HPO, which contains sentence-level annotations with two entity types, and Occup-Sub, which provides document-level annotations across six entity categories. We compared TriG-NER and DocDiscNER across both datasets and analysed their performance across several NER attributes, including sentence length, entity density, and out-of-vocabulary density. We also assessed computational cost, evaluated the integration of each model with an NEN component, examined GPT-4.1 in a few-shot setting for this task, and conducted a qualitative error analysis.

**Results:**

TriG-NER achieved the best performance on BioCreative-HPO, with an F1 score of 78.2%, while DocDiscNER performed best on Occup-Sub, with an F1 score of 82.2%. These results demonstrate the effectiveness of TriG-NER in sentence-level contexts and the advantage of DocDiscNER in longer, document-level contexts involving multiple entity categories. TriG-NER also showed superior computational efficiency, requiring less training time and GPU memory. In contrast, DocDiscNER benefited from the Coordination Ellipses Resolution (CER) component, which improved its handling of complex discontinuous structures. Despite its potential, GPT-4.1 underperformed in the few-shot setting.

**Discussion:**

The findings highlight complementary strengths between grid-tagging and generative approaches for DiscNER in healthcare. TriG-NER is more computationally efficient and performs strongly in sentence-level settings, whereas DocDiscNER is better suited to longer and more complex document-level contexts. The limited performance of GPT-4.1 suggests that full fine-tuning or task-specific adaptation may be necessary to achieve optimal performance in DiscNER.

## 1 Introduction

Named Entity Recognition (NER) has been widely applied across biomedical, clinical, and general-domain texts to identify Named Entities (NEs) of interest such as names, dates, places, and medical terms. Traditional NER approaches typically assume that entities appear as continuous spans of text. However, clinical and biomedical datasets often contain Discontinuous Named Entities (DiscNEs)—entities that are split across non-adjacent tokens but still form a single semantic unit [1, 2]. For example, the expression “arm and elbow pain” contains two entities, one of which (“arm pain”) is formed by non-contiguous tokens. In healthcare texts, approximately 9% to 14% of entity spans are discontinuous [3–5]. This presents a significant challenge, as sequence labelling methods often struggle to recognise and normalise such fragmented entities [6]. Accurate identification of DiscNEs is critical for downstream biomedical tasks such as Named Entity Normalisation (NEN) and relation extraction, where undetected or improperly delineated DiscNEs can lead to incomplete or incorrect interpretations [7].

In recent years, several methods have been proposed to address the challenges posed by DiscNEs. A recent systematic review of DiscNER methods [1] consolidated per-method performance on the three established DiscNER benchmarks, CADEC, ShARe-13, and ShARe-14, across all major approach paradigms. Based on this review and the subsequent release of TriG-NER [8] and DocDiscNER [9], both models report new SOTA results on at least one of the three benchmarks under the standardised evaluation script of Dai et al. [2]. Beyond their performance, the two systems instantiate fundamentally different architectural paradigms. TriG-NER employs a grid-based architecture [10, 11] to capture word-pair relationships using a flexible grid structure. It adapts triplet loss to model local dependencies between tokens, making it well suited for identifying DiscNEs. In comparison, DocDiscNER leverages generative large language models (LLMs) and document-level contextual representations, incorporating two auxiliary components, namely Coordination Ellipses Resolution (CER) and self-consistency decoding [12], to enhance entity recognition.

As these two models substantially outperform earlier discontinuous NER baselines, including MAC [13], TOE [11], BARTNER [14], W${}^{2}$NER [10], and Corro [15], they provide a strong basis for comparative analysis. We therefore systematically compare these approaches, focusing on their strengths, limitations, and generalisability across datasets with diverse entity types. We also assess their practical performance when integrated with an NEN component. Additionally, we provide a structured evaluation of GPT-4.1 in a few-shot setting on DiscNER tasks to assess their ability to identify DiscNEs compared to specialised models.

This paper aims to address the following research questions:

1. How does each model compare in terms of performance, efficiency, and associated computational costs?
2. How effectively do these models generalise across datasets from various domains?
3. How does their performance vary when integrated with an NEN component?

The key contributions of this paper are:

- A systematic comparison of two SOTA DiscNER approaches: DocDiscNER and TriG-NER, focusing on their methodologies, strengths, and limitations across multiple datasets.
- A comprehensive error analysis of NER and NEN outcomes, identifying the main challenges encountered by both models, identifying recurring failure patterns, and outlining potential directions for improvement.
- A comparative evaluation of GPT 4.1 in few-shot settings, assessing its ability to recognise DiscNEs relative to task-specific models.

Our comparative analysis shows that TriG-NER performs better on datasets composed of individual sentences with limited entity types, as its graph-based approach effectively models local dependencies. In contrast, DocDiscNER demonstrates superior performance on document-level datasets, where a broader context and a greater variety of entities are present. Meanwhile, GPT-4, despite its generalisation capabilities, struggles with DiscNE recognition, particularly in complex clinical texts, due to the lack of explicit structural modelling offered by specialised NER architectures.

The remainder of this paper is structured as follows: Section 2 reviews related work. Section 3 describes the DocDiscNER and TriG-NER methodologies, along with our experimental setup. Section 4 presents the results and performance patterns. Finally, Section 5 presents a detailed discussion and in-depth error analysis, with key insights that can benefit future research.

## 2 Related work

Existing work in the NER literature has explored DiscNER, with significant advancements driven by novel deep learning architectures, alternative tagging schemes, and generative paradigms. In this section, we outline key approaches to DiscNER, highlight major corpora annotated with DiscNEs, and review comparative studies relevant to NER performance evaluation.

### 2.1 Approaches to DiscNER

NER has historically been treated as a sequence labelling task that assumes entities appear as continuous spans. However, this assumption fails to capture more complex structures such as discontinuous entities, which has prompted a range of research efforts focused on developing more flexible approaches. Early solutions extended traditional tagging schemes; for instance, Tang et al. [16] proposed modifications to the BIO tagging format through schemes like BIOHD and BIOHD1234 to enable the recognition of split and overlapping entities. Transition-based models such as the one introduced by Dai et al. [2] utilised shift-reduce parsing strategies, which performed well on some discontinuities but struggled with overlapping cases due to inherent structural limitations in tag representation. Span-based methods were also explored by Fei Li et al. [17], who introduced a strategy that involves enumerating all possible candidate spans. While this method achieved reasonable performance, it also led to significant computational overhead due to its combinatorial nature.

In parallel, hypergraph-based approaches modeled non-contiguous structures with shared subcomponents. Methods proposed by Muis and Lu [18] and by Wang and Lu [19] demonstrated the ability to identify DiscNEs using hypergraph representations, though their decoding complexity limited scalability in large-scale applications. A more recent method involves grid-tagging architectures, which map token relationships onto a 2D grid. Li et al. [10] introduced a model incorporating relation types such as Next-Neighboring-Word and Tail-Head-Word, and this framework was further enhanced by Liu et al. [11] through the addition of two more relations: Previous-Neighboring-Word and Head-Tail-Word, leading to richer entity boundary modelling. Building on this line of work, TriG-NER [8] adopted a grid-based formulation with a triplet loss function that models both token-level and entity-level co-occurrence, resulting in improved performance across varying complexities of DiscNEs.

In contrast to these tagging-heavy paradigms, generative approaches have emerged as a more flexible alternative. These models, typically based on sequence-to-sequence architectures, generate entity mentions directly as output sequences, removing the dependency on pre-defined tag sets. Several recent studies [14, 20, 21] have demonstrated the viability of this approach for handling complex entity structures, including nested and discontinuous spans. DocDiscNER [9] builds on this paradigm by incorporating a generative LLM with two auxiliary mechanisms: Coordination Ellipses Resolution (CER) and self-consistency decoding, within a document-level context, significantly enhancing its ability to capture long-range dependencies and fragmented mentions. In this work, we compare these two leading approaches—TriG-NER and DocDiscNER—representing the grid-tagging and generative paradigms respectively, to provide insight into their relative strengths and limitations.

### 2.2 Corpora annotated with DiscNEs

While many NER corpora are not annotated for DiscNEs, several specialised datasets in the biomedical and clinical domains have incorporated such annotations driven by the need for more precise clinical or biomedical entities to better support downstream tasks. Among the most widely annotated DiscNER corpora are ShARe-13 [3] and ShARe-14 [22], which were released as part of the ShARe/CLEF shared tasks and have become benchmarks for evaluating DiscNER performance in clinical texts. These datasets include a range of challenging DiscNEs that frequently occur in patient records. Another benchmark corpus is CADEC [4], which focuses on adverse drug reactions (ADRs) collected from user-generated content. It includes non-contiguous spans reflecting real-world variability in how patients express medical conditions.

The BioCreative shared task [5] further expanded the scope of DiscNER by introducing a dataset that captures phenotypic mentions in paediatric genetic conditions, aligned with the Human Phenotype Ontology (HPO) [23]. This dataset contains a high proportion of DiscNEs (up to 14%) and provides normalised concept annotations mapped to the HPO. Thompson et al. [24] introduced a new annotated corpus centred on occupational substance exposure, drawn from scientific literature. This dataset includes six entity types, such as exposure measured and occupation, expanding the scope of DiscNER beyond traditional biomedical applications. These two recent corpora, which encompass a range of domains, annotation practices, and linguistic challenges, present valuable opportunities for further evaluation of DiscNER methods, and have therefore been selected for this study.

### 2.3 Comparative studies in NER

Prior work has mostly compared the performance of pre-trained language models for NER across both general-domain and domain-specific datasets. For example, Li et al. [25] investigated how various BERT-based NER models perform on clinical trial eligibility texts. Their results showed that domain-specific transformers consistently outperformed general-domain ones. In another effort, Liu et al. [26] benchmarked seven NER models, including both deep learning and transformer-based models, on three medical datasets. Their analysis revealed that entity length and phrase complexity were key factors influencing model performance, pointing to a need for finer-grained evaluation metrics. In a multilingual setting, Ozcelik et al. [27] conducted an empirical comparison of machine learning, deep learning, and Transformer-based models across five Turkish NER datasets. They concluded that Transformer-based approaches were more robust in scenarios involving long or sparsely occurring entities. Finally, complementing these studies, Fu et al. [28] proposed an interpretable evaluation framework for NER, which examined eight attribute types—such as entity length, ambiguity, and lexical variability—to understand how they correlate with changes in F1-scores across models.

Despite these efforts, no existing work has systematically compared DiscNER models across multiple corpora with varying entity types and domain characteristics. While the above studies contain valuable insights into NER models’ robustness and evaluation strategies, they do not directly compare performance of different DiscNER modelling paradigms. In response to this gap, our study provides an in-depth evaluation of DocDiscNER and TriG-NER, two state-of-the-art DiscNER models, using datasets from the healthcare domain. Furthermore, we investigate how these models perform when integrated with an NEN component, providing an end-to-end perspective on their practical utility.

## 3 Material and methods

### 3.1 Models overview

This study presents a comparative analysis of two of the SOTA models for DiscNER. Specifically, we implement two existing models and evaluate their effectiveness across two datasets spanning multiple health domains, entity types, and span complexities. Our systematic evaluation, based on key performance metrics, highlights the strengths and limitations of each model in capturing DiscNEs.

To the best of our knowledge, these two models represent the highest-reported performance on the three standard DiscNER benchmarks. DocDiscNER [9] establishes new SOTA on CADEC and ShARe-14, surpassing the previous best by 2.48 [20] and 1.56 [14] absolute F1 points, respectively, while TriG-NER [8] establishes new SOTA among grid-tagging approaches [10, 11, 13] on all three benchmarks [3, 4, 22]. Both models also outperform prior baselines specifically on the recognition of discontinuous mentions, which is the focus of our analysis. Despite reaching comparably high performance, the two systems are built on fundamentally different paradigms: TriG-NER is a discriminative, sentence-level grid-tagging framework, whereas DocDiscNER is a generative, document-level LLM-based framework. This architectural difference makes the two models a well-suited pair for comparative analysis, as it helps guide model selection across different NER deployment scenarios.

The following sections provide a detailed discussion of each model.

#### 3.1.1 TriG-NER

The Triplet-Grid NER framework [8] is an innovative approach that builds upon the grid matrix scheme and extends two prior grid-tagging architectures: W2NER [10] and TOE [11]. Unlike previous grid-tagging methods that treat each word pair independently, TriG-NER models word-pair relationships within a flexible grid structure, where token pairs (rather than individual tokens) are explicitly labelled. In this scheme, positive pairs represent tokens that belong to the same entity, while negative pairs consist of word pairs disrupted by non-entity tokens. This structure forms an extended enhancement over conventional grid-tagging architectures.

A distinctive feature of this framework is the use of a triplet loss learning objective at the word-pair level, enabling the model to learn fine-grained distinctions between similar and dissimilar word pairs within the grid. The grid-tagging classifier assigns word-pair relationships to one of three tag classes: None, Next-Neighboring-Word (NNW), and Tail-Head-Word (THW). These categories indicate whether a word pair has no association, forms a neighbouring connection within the same entity, or marks the start and end of an entity, respectively. This tagging strategy allows the model to effectively capture non-contiguous entity spans.

To further optimise performance, the framework incorporates the triplet mining method, where candidate triplets are derived either from the Word-Pair Relationship matrix or the final output logits. Four triplet selection strategies are employed: Hard Negative, Semi-hard Negative, Centroid, and Negative Centroid. Through these mechanisms, the model achieves substantial performance gains, outperforming existing SOTA grid-based architectures, particularly in recognising DiscNEs.

#### 3.1.2 DocDiscNER

Document-level Discontinuous NER [9] is a recent approach that leverages the broader context of a text document—rather than isolated sentences—as input, which has been shown to enhance entity recognition. To handle long documents efficiently, it employs a semantic chunking algorithm, grouping sentences only when their similarity score meets a predefined threshold.

The model adopts a generative LLM as its backbone, removing the reliance on traditional labelling schemes [6, 15]. Instead, it takes as input the raw textual spans with their corresponding entity types and directly generates the named entity tokens. This design enables a more generalisable and flexible solution across different NER scenarios.

The architecture incorporates two auxiliary components. The first is Coordination Ellipses Resolution, which identifies coordination ellipsis expressions—sentences where two or more items are joined by coordinating conjunctions (e.g., and, or)—and expands them by adding the missing heads. This ensures that all coordinated named entities are accurately captured.

The second component is the Span-Level Self-Consistency decoding method, which acts as an optimiser for the generated outputs. It samples multiple reasoning paths from the decoder using beam decoding, generating potential span candidates. The final output is selected based on whether it appears at least k times across the generated paths, enhancing output reliability. This model achieves SOTA performance, particularly in the recognition of discontinuous mentions.

**Figure 1:**
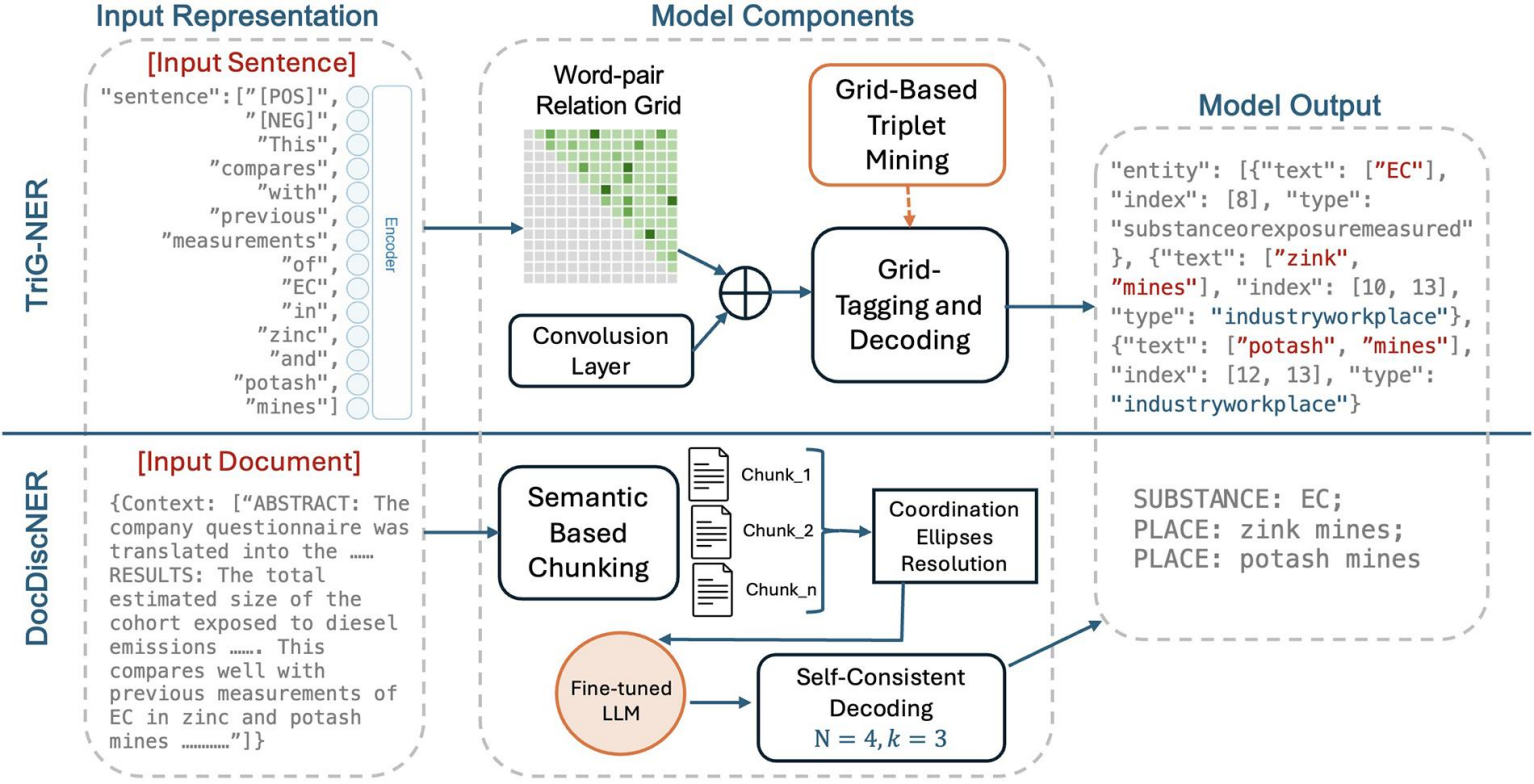
TriG-NER and DocDiscNER: model architectures. The TriG-NER architecture is adapted from “Overall framework of the proposed TriG-NER” by Rina Carines Cabral, Soyeon Caren Han, Areej Alhassan, Riza Batista-Navarro, Goran Nenadic and Josiah Poon, licensed under CC BY. The DocDiscNER architecture is adapted from “DocDiscNER model architecture” by Areej Alhassan, Viktor Schlegel, Rina Carines Cabral, Riza Batista-Navarro, Soyeon Caren Han, Josiah Poon and Goran Nenadic, licensed under CC BY-NC.

Figure 1 illustrates both the TriG-NER and DocDiscNER architectures, along with their respective input and output representation schemes.

### 3.2 Datasets

Since the two models have so far been tested only on DiscNER benchmark datasets: ShARe-13 [3], ShARe-14 [22], and CADEC [4], where each contains only a single entity type, we sought to examine their generalisability across datasets with a greater variety of entity types and context levels. For this purpose, we selected two recent corpora in which DiscNEs are explicitly annotated.

The first dataset, BioCreative-HPO [5], consists of independent sentences annotated with two entity types. The second dataset, the Occupational Substance Exposure (Occup-Sub) dataset [24], contains full documents annotated with six entity types, making it well-suited to evaluate the models’ capabilities in document-level settings with higher entity diversity. Dataset statistics are summarised in Table 1.

**Table 1:** Statistical analysis of the BioCreative-HPO and occupational exposure corpora.

Analysis	BioCreative_HPO			Occupational_Exposure			
	All	Training	Valid	All	Train	Valid	Test
No. Sentences	2,170	1,716	454	12,827	10,261	1,283	1,283
No. Entities	3,247	2,562	685	16,203	13,170	1,492	1,541
No. Disjoint Mentions	465	369	96	748	606	74	68
Percent of Disjoint mentions	14.3%	14.4%	14%	4.62%	4.5%	5%	4.4%
DiscNEs (2-constituent parts)	449	358	91	726	590	69	67
DiscNEs (3-constituent parts)	16	11	5	20	15	4	1
DiscNEs More than 3 parts	0	0	0	2	1	1	0
DiscNEs with shared heads	408	325	83	290	238	31	21
DiscNEs with no shared heads	57	44	13	458	368	43	47

The test set of BioCreative-HPO is not publicly available; therefore, its statistics are not reported.

During preliminary experiments, we observed noise and inconsistencies in both datasets. As a result, we made minor modifications and used our own processed versions in the evaluation. In the following section, we describe each dataset in detail, including their key characteristics and any adjustments made to ensure consistency and quality.

#### 3.2.1 BioCreative-HPO

**Figure 2:**
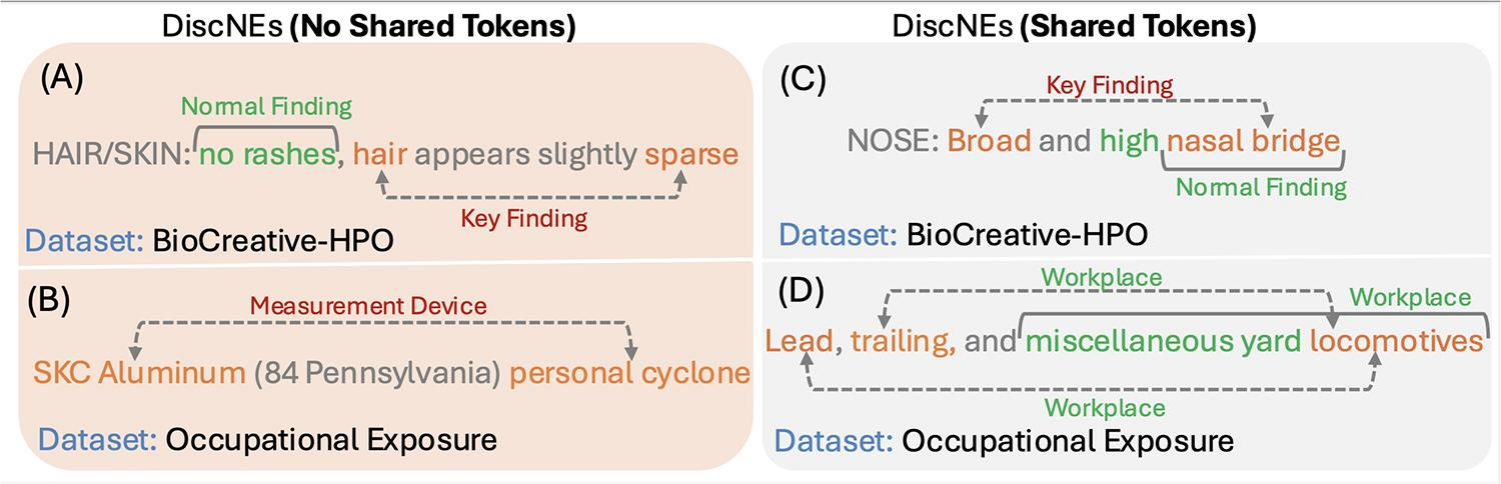
Examples of DiscNEs in both datasets. Dotted arrows indicate DiscNEs, whereas solid arrows represent continuous mentions.

This dataset was developed for the genetic phenotypic extraction and normalisation task, focusing on dysmorphology and physical examination observations in paediatric patients. It was introduced as part of BioCreative VIII—Task 3 [5], which involves recognising key phenotypic findings while disregarding normal observations, and then mapping these findings to the HPO [23]. The dataset consists of 3,136 de-identified and manually annotated observations extracted from the electronic health records of 1,652 paediatric patients at the Children’s Hospital of Philadelphia. Each observation is presented as a single sentence, beginning with the examined organ (see Figures 2A,C).

The corpus defines two entity types based on the clinical polarity of findings in dysmorphology physical examinations. Key findings denote abnormal phenotypes potentially suggestive of an underlying genetic condition and are the primary targets of the normalisation task, each mapped to a corresponding HPO term. Normal findings describe observations that are noted by clinicians to rule out specific disorders. Since the HPO lacks terms for normal phenotypes, these are normalised to their closest corresponding abnormal HPO term and marked as Negated. Only key findings are considered medically relevant for downstream genetic analysis.

A defining characteristic of this dataset is its high proportion of DiscNEs, which account for 14% of the annotations in the training and validation sets and 16.9% in the test set, which is the highest proportion among existing English DiscNER corpora [1]. Sentences often contain multiple DiscNEs of varying complexity, making this dataset particularly valuable for evaluating DiscNER models. The frequent occurrence of disjoint mentions primarily results from the high prevalence of coordinated expressions, which account for 87.7% of all DiscNEs. Another contributing factor is the annotation strategy, which aims to include only the minimum necessary tokens for accurate ontology mapping. For example, in the phrase “hair appears slightly sparse”, only “hair” and “sparse” are annotated to ensure correct alignment with the HPO term “Sparse hair (HP:0008070)”. According to the annotation guidelines, only the essential terms conveying the meaning of the normalised HPO concept should be included, while qualifiers such as “slightly” or “potentially” are excluded to maintain clarity and consistency in ontology mapping.

For our experiments, we used the same training, validation, and test splits provided by the task organisers. The numbers of sentences and annotated spans are shown in Table 1, while the detailed named entity counts per class are provided in Table 2. For the test set, it is important to note that the organisers released only the unannotated sentences. Evaluation can therefore only be performed via the CodaLab platform, which provides aggregate scores without granting access to the gold-standard annotations. As a result, NER results on the test set cannot be computed or reported; instead, all NER evaluations in this work are conducted on the validation set.

**Table 2:** Per entity class count of the BioCreative-HPO and Occup-Sub corpora, and DiscNEs count per class (percentage per class is within the brackets).

Dataset	Entity type	NEs per class			DiscNEs per class		
		Training	Validation	Test	Training	Validation	Test
BioCreative-HPO	Key Finding	2,233	607	–	274 (12.3)	79 (13.0)	–
	Normal Finding	329	78	–	95 (28.9)	17 (21.8)	–
Occup-Exp	Exposure Measured	6,558	641	716	171 (2.6)	18 (2.8)	19 (2.7)
	Sample Type	418	41	72	35 (8.3)	2 (4.9)	12 (16.7)
	Industry/Workplace	2,255	296	338	107 (4.7)	10 (3.4)	4 (1.2)
	Occupation/Job Title	1,820	219	180	100 (5.5)	6 (2.7)	10 (5.6)
	OH Device	671	129	132	18 (2.6)	3 (2.3)	4 (3.0)
	Job Task/Activity	1,448	166	103	178 (12.3)	35 (21.1)	21 (20.4)

#### 3.2.2 Occup-Sub

The Occupational Substance Exposure (Occup-Sub) corpus [24] was primarily developed to study both existing and emerging substance exposures in working environments, recognising that occupational exposures are major contributors to non-communicable diseases. It was created by annotating exposure-related scientific literature that mentions workplace substance exposures, as part of the Exposome Project for Health and Occupational Research (EPHOR). Extracting such entities facilitates the analysis and interpretation of current exposure threats (e.g., toxic chemicals, hazardous dusts, industrial fumes), thereby supporting the development of improved workplace health measures and safety protocols.

This dataset is annotated with six entity types:

- Industry/Workplace: Terms referring to an economic activity, e.g., “diesel factory” or “the construction of buildings”.
- Occupation/Job Title: Roles or positions of individuals or groups, e.g., “electricians” or “carpenters”.
- Job Task/Activity: Specific physical activities carried out as part of daily work duties, e.g., “welding” or “concrete pouring”.
- Substance or Exposure Measured: Names of substances, chemicals, or pollutants that are measured or sampled, e.g., “elemental carbon” or “diesel exhaust”.
- Occupational Hygiene (OH) Measurement Device: Devices or tools used to measure particulate and gaseous exposures in the workplace, e.g., “Higgins Dewell cyclones”, “large cyclone separator”, or “real-time particle exposure monitors”.
- Sample Type: Personal: Phrases indicating that collected airborne substance samples represent personal exposures, e.g., “personal breathing zone sample” or “personal full-shift samples”.

The occurrence of DiscNEs in this dataset primarily caused by two situations: (1) the presence of coordination ellipsis expressions, (2) the annotation of DiscNEs where bracketed information (such as explanatory terms or abbreviations) interrupts the entity span. In the second case, discontinuous annotations are used to exclude the bracketed content that appears mid-phrase (see Example B in Figure 2). For our experiments, we followed the evaluation split of the corpus creators, using an 80/10/10 split for training, validation, and testing, respectively.

### 3.3 Attribute-based NER evaluation

We follow the work by Fu et al. [28] which investigates how specific dataset characteristics influence NER performance across all NEs. The goal of this evaluation is to examine how different models behave under varying conditions, as these factors can guide the selection of suitable architectures depending on the dataset’s properties. To this end, we analyse model predictions with respect to three dataset-specific attributes previously identified as impactful: input sentence length (sLen), entity density (eDen), and out-of-vocabulary density (oDen). These three analyses were performed using input sentences from BioCreative-HPO and the original sentence-level splits of the Occup-sub dataset, along with the corresponding NER predictions from DocDiscNER and TriG-NER. The following sections define these attributes.

#### 3.3.1 Input length

To analyse model performance across varying input sizes, we group input instances based on their length, defined as the number of words in each instance. For each length group, we compute the corresponding F1 score, enabling a comparison of performance across different input length ranges.

#### 3.3.2 Entity density

This attribute measures how many named entities a sentence contains per unit length. We examine how both models perform across sentences with different densities. The entity density $\phi_{\text{eDen}}(x)$ of a sentence x is defined as:

$$\phi_{\text{eDen}}(x)=\frac{\left|\text{ent}(\text{sent}(x))\right|}{\phi_{\text{sLen}}(x)}$$

where:

- sent(x): Represents an input sentence x.
- ent(sent(x)): The set of named entities found in the sentence x.
- |ent(sent(x))|: The number of named entities in the sentence (i.e., the cardinality of the entity set).
- $\phi_{\text{sLen}}(x)$: The length of the sentence, typically measured in words or tokens.

#### 3.3.3 Out of vocabulary density

Out Of Vocabulary (OOV) density measures the proportion of words in an input sentence that are not present in the predefined training vocabulary. It reflects how unfamiliar or unseen a sentence is to the model based on its training data. To compute the OOV density ($\phi_{\mathrm{oDen}}(x)$), we first require the training vocabulary V, which represents the set of unique words observed in the training data.

The OOV density for a sentence x is defined as:

$$\phi_{\mathrm{oDen}}(x)=\frac{\left|\text{oov}(\text{sent}(x))\right|}{\phi_{\text{sLen}}(x)}$$

where:

- oov(sent(x)): the set of words in the input sentence x not found in the training vocabulary.
- |oov(sent(x))|: the count of words in the input sentence x that do not appear in the training vocabulary.
- $\phi_{\text{sLen}}(x)$: the *sentence length*, typically measured in the number of words.

For both examined densities in Sections 3.3.2 and 3.3.3, we bin entity density into equal-width ranges of 0.05 (e.g., [0,0.05], [0.05,0.10], [0.10,0.15]) and plot each bin at its midpoint (0.025, 0.075, 0.125, 0.175, …). These finer-grained density intervals provide a more detailed view of how model performance varies across different levels of entity densities while still covering the majority of input sentences in both corpora. In addition, For each density bin, we computed sentence-level F1 scores for each model and then calculated the mean F1 across all sentences within that bin. The result was visualised as a line plot, where the x-axis represents the midpoint of each entity-density bin and the y-axis represents the mean F1 score. Error bars correspond to 95% confidence intervals estimated from the sentence-level F1 scores in each bin.

#### 3.3.4 DiscNEs-based attributes

To examine the specific model performance on DiscNEs, we conduct a structured evaluation across two dimensions. First, we examine length-related properties, including interval length and overall span length of DiscNEs. Second, we analyse structural variations by categorising DiscNEs into overlapping and non-overlapping entities.

##### 3.3.4.1 Interval and span length

Interval length refers to the number of words separating the discontinuous parts of a mention, while span length represents the total word count of the full mention, including intervening tokens that are not part of the named entity. We follow the evaluation framework of Wang et al. [13] to compare the ability of both models to recognise DiscNEs across varying interval and span lengths. To facilitate interpretation of the corresponding analysis, Table 3 additionally reports the number of DiscNE instances in each interval-length and span-length category.

**Table 3:** Count of DiscNEs in each dataset (validation set of BioCreative-HPO, test set of Occup-Sub), grouped by the length of interrupting tokens (*Interval length* or Intv) and the total length of the discontinuous span, including interrupting tokens (*Span length*).

Length	BioCreative-HPO		Occup-Sub	
	Intv	Span	Intv	Span
l=1	14	N/A	4	N/A
l=2	30	N/A	35	N/A
l=3	12	5	15	3
l=4	9	18	4	24
l=5	9	15	5	15
l=6	8	18	3	9
l≥7	8	34	2	17

Spans of lengths 1 and 2 are marked as N/A since they cannot form discontinuous spans.

##### 3.3.4.2 Discontinuous structures

To evaluate our model’s ability to extract mentions with shared heads, we refer to previous studies that classify DiscNE structures as overlapping and non-overlapping [2, 13]. DiscNEs can be categorised into four overlapping types: (1) no overlap, (2) left overlap, (3) right overlap, and (4) multiple overlap. Figure 2 provides examples of both overlapping and non-overlapping cases.

### 3.4 Integration with named entity normaliser

NEN is the process of mapping identified NEs to standardised concepts within a structured ontology. This step ensures that medical terms correspond to the closest standardised concept, thereby facilitating consistency in clinical and biomedical text processing. In this study, our aim was to compare and assess whether the entity spans extracted by different NER models result in different normalised outputs when processed using a fixed normaliser. Specifically, we examined how the normalised outputs of TriG-NER and DocDiscNER differ, and we identified common normalisation errors shared by both models.

The BioCreative-HPO dataset includes normalisation annotations, mapping both key findings and normal findings to their corresponding HPO identifiers. We utilised these gold-standard annotations for our comparison. First, we fine-tuned multiple large language models (LLMs) on an augmented list comprising 12,000 HPO terms and their synonyms, derived from the training data. We then evaluated the models on the normalised gold-standard entity spans from the BioCreative-HPO validation set. After benchmarking their performance, we selected the best-performing LLM to serve as our normalisation component. Finally, we applied this normaliser to the predicted entity spans from TriG-NER and DocDiscNER, enabling a systematic evaluation of how effectively each model’s extracted entities can be mapped to standardised ontology concepts.

### 3.5 Experimental settings

#### 3.5.1 Preprocessing

For both datasets, we began by addressing several annotation inconsistencies that could negatively impact extraction performance. In BioCreative-HPO, minor inconsistencies were identified in both the training (3 cases) and validation (4 cases) sets. We resolved these issues and used the corrected versions for our experiments. In Occup-Sub, inconsistencies arose mainly from sentence segmentation, where some NEs spanned across two separate sentences. To address this, we merged sentences containing multi-sentence entities (24 instances in total). We also corrected 47 cases where white space was mistakenly omitted between a token and an adjacent named entity—either due to tokenisation errors or inconsistencies in the annotation platform. A detailed list of these adjustments is included in Supplementary Tables S1 and S2 in the Appendix.

Both datasets were originally provided as sentences with their corresponding entity annotations. In BioCreative-HPO, each sentence represents an independent and self-contained medical examination entry. Although Occup-Sub was already segmented into sentences, merging them may provide richer context for document-level processing [9]. Therefore, we combined all sentence-level instances belonging to the same scientific article to form a single document. We then applied semantic chunking (also known as embedding-based chunking) to split each document into semantically coherent paragraphs, ensuring an optimal sequence length while preserving sufficient contextual information.

At the input level, for TriG-NER, we retained the original sentence-level format for both datasets. For DocDiscNER, we used sentence-level instances of the BioCreative-HPO corpus and the document-level version of the Occup-Sub corpus. As shown in Figure 1, the input format differs by model. TriG-NER follows the conventional NER input representation, where each input instance corresponds to a single sentence, encoded as a sequence of words and special tokens that are independently processed by the encoder. In contrast, DocDiscNER employs a free-form textual input format consistent with the generative nature of LLMs: text chunks are provided as input, and numerical offsets for each NE are converted into their corresponding textual spans. Each NE is prefixed with its entity type, for example, “KEYF:” for key findings in BioCreative-HPO, and “SUBSTANCE:” for substances or exposures measured in Occup-Sub.

#### 3.5.2 Few-shot prompting setup for NER

To assess the capability of LLMs in recognising both continuous and discontinuous named entities under a few-shot prompting strategy, we structure each prompt to comprise a task description, followed by definitions of all entity classes used in the dataset. For each class, representative examples are provided to guide the model’s interpretation of entity boundaries and types. A predefined output format is specified to ensure consistency and facilitate evaluation. We consider three few-shot configurations: 2-shot, 4-shot, and 6-shot. In the 2-shot setting, only continuous entity examples are included. The 4-shot configuration introduces one example containing an overlapping discontinuous named entity (DiscNE), while the 6-shot configuration includes two DiscNE examples, covering both overlapping and non-overlapping structures. This allows us to examine the impact of progressively introducing discontinuous patterns into the prompt context. All prompts are applied consistently across the evaluation dataset. The complete prompt templates and corresponding examples for each configuration are provided in Supplementary Section S2 of the Appendix.

#### 3.5.3 Implementation details

##### 3.5.3.1 TriG-NER

For the encoder layer, we fine-tuned several popular Pre-trained Language Models (PLMs), including BioBERT [29], BioClinicalBERT [30], PharmBERT [31], and PubMedBERT [32]. The best-performing configuration for both datasets was PubMedBERT. The model was trained using the AdamW optimiser with a learning rate of 5e-4, a batch size of 16, and a maximum of 60 epochs, with an early stopping criterion of 10 epochs. We adopted the grid-centroid triplet mining strategy, which outperformed other triplet mining methods (including hard-negative, semi-hard negative, and negative centroid) in effectively capturing both continuous and discontinuous spans. In addition, the optimal triplet source differed across datasets: the Word-Pair Relationship Grid ($H_{bi}$) yielded the best performance on BioCreative-HPO, whereas the final grid-tag logits (Y) were more effective for Occup-Sub. All TriG-NER experiments were conducted on a single NVIDIA RTX A4500 GPU.

##### 3.5.3.2 DocDiscNER

For the backbone generative LLM, we experimented with several models, including Mistral-7B, BioMistral-7B [33], and LLaMa3-8B [34]. For the BioCreative-HPO dataset, BioMistral-7B was the optimal choice, leveraging its medical-domain pretraining, while LLaMa3-8B achieved the best results for the Occup-Exp dataset, outperforming the other tested LLMs. The model was trained using the AdamW optimiser with a batch size of 8 and a learning rate of 5e-4, applying a linear learning rate scheduler. Training lasted for 20 epochs. To improve efficiency, we incorporated LoRA [35] with r=256 and α=128, enabling memory-efficient fine-tuning. All DocDiscNER experiments were conducted on a single NVIDIA A100 GPU.

It should be noted that the backbone and configuration selection was not fixed for either model, but was determined empirically based on the performance for each dataset. This follows the evaluation principle used in the original TriG-NER [8] and DocDiscNER [9] architectures, where multiple PLMs or LLMs were compared and the best-performing setup for each dataset was reported. In our study, TriG-NER was evaluated with several encoder backbones, and PubMedBERT achieved the best results on both BioCreative-HPO and Occup-Sub; similarly, for DocDiscNER, BioMistral-7B was optimal for BioCreative-HPO, whereas LLaMA3-8B performed best on Occup-Sub. This strategy was applied consistently to both models and was intended to compare their strongest validated configurations.

##### 3.5.3.3 Consistent computational setup

To enable a fair efficiency comparison between the two models, which were originally trained under different computational settings, we re-trained both DocDiscNER and TriG-NER on an NVIDIA L4 GPU (24GB), an energy-efficient GPU optimised for lightweight training and inference tasks.

##### 3.5.3.4 Few-shot NER

GPT-4.1 was accessed via the OpenAI Chat Completions API using the model identifier gpt-4.1-2025-04-14. Inference was conducted on Google Colab. Each API call was formulated as a multi-turn interaction, consisting of a system prompt defining the task, followed by user and assistant turns providing few-shot examples, and a final user turn containing the target sentence. To promote stable and reproducible outputs, we set the generation temperature to 0.01 and generated a single response per request. The maximum token limit was not explicitly constrained beyond the API defaults. Although recent API versions provide a seed parameter (set to 1 in our experiments), deterministic behaviour is not strictly guaranteed, and model outputs may still produce minor variability across runs. While we observed qualitatively consistent outputs, we acknowledge that repeated executions may yield slight variations; incorporating multiple runs and reporting variance measures is left for future work.

##### 3.5.3.5 NEN component

For the NEN module, we used Llama-3-8B-Instruct, integrating FlashAttention-2 [36] to accelerate training, particularly when processing the extensive HPO dictionary. To ensure compatibility with the “instruct” model variant, we formatted the input prompts using the chat template structure, which includes user, system, and assistant roles. The LoRA configuration was consistent with DocDiscNER (r=256, α=128). The model was trained for 20 epochs with a batch size of 8 and a learning rate of 5e-4. All NEN experiments were conducted on a single NVIDIA A100 GPU.

#### 3.5.4 Evaluation metrics

For NER, we report span-level (micro-averaged) precision, recall, and F1 scores. A predicted entity is counted as a true positive (TP) if its span exactly matches a gold-standard entity, while a false positive (FP) corresponds to a predicted span that does not match any gold entity, and a false negative (FN) denotes a gold entity that was not predicted. Precision is computed as TP/(TP + FP), recall as TP/(TP + FN), and F1 as the harmonic mean of precision and recall. For DiscNER We specifically follow Wang et al. [13] and Dai et al. [2] in adopting two evaluation schemes: (1) Reporting the results for sentences that include discontinuous mentions by considering both the extracted continuous and discontinuous named entities (NEs) present in the same sentence. (2) Reporting results for discontinuous mentions only, calculating extraction performance exclusively on DiscNEs while excluding continuous mentions within the same sentence.

For the NEN component on the BioCreative-HPO dataset, we follow the BioCreative VIII Task 3 evaluation protocol [5] using three settings. In the normalisation-only setting, performance is measured by comparing predicted HPO term IDs against the gold standard at the observation level using precision, recall, and F1-score. In the exact extraction and normalisation setting, a prediction is considered correct only if both the entity span and the assigned HPO term exactly match the reference. In the partial (overlapping) extraction and normalisation setting, predictions are counted as correct when the predicted span overlaps with the gold span and the HPO term is correct.

#### 3.5.5 Baselines

In addition to comparing the performance of our two models, we evaluate them against established baselines that have used the same datasets in their original evaluations. For BioCreative-HPO, We compare our NER results against the NER component of the DiscHPO pipeline [37], which employs a generative approach based on the Flan-T5 [38] model. This model produced detailed DiscNE results, making it a suitable baseline for our evaluation.

For Occup-Sub, we compare our results with the span-based NER baseline introduced in the original corpus paper [24]. In this approach, all possible spans within a sentence, comprising varying numbers of tokens, are enumerated. The model includes an information compression mechanism and two unsupervised components: span reconstruction and synonym generation using external knowledge bases.

For the NEN baselines, we compare against three HPO normalisers:

##### 3.5.5.1 DiscHPO normaliser [37]

This component follows a sentence-transformer framework, incorporating a bi-encoder for candidate generation and a cross-encoder for reranking. The top-scoring candidate is selected as the final output.

##### 3.5.5.2 Phenotagger [39]

A hybrid system that combines an HPO dictionary with machine learning, leveraging several PLMs, including Bioformer [40], BioBERT, and PubMedBERT. It achieved SOTA performance on HPO-annotated corpora such as JAX and GSC+ [41]. For our comparison, we used the results reported by Weissenbacher et al. [5] on the BioCreative VIII HPO dataset, specifically the version based on BioBERT.

##### 3.5.5.3 PhenoID [42]

This system follows a two-phase architecture. In the first phase, it performs NER using a BIO tagging scheme with BioClinicalBERT as the underlying PLM. In the second phase, normalisation is treated as a classification task, where BioClinicalBERT is fine-tuned to predict a probability distribution over 11,988 HPO concept IDs.

## 4 Results

### 4.1 NER results

In Table 4, we first report the NER results of BioCreative-HPO on the validation set.

**Table 4:** NER results for the BioCreative-HPO validation set, including both overall and per-class scores.

Category	Baseline (DiscHPO)			TriG-NER			DocDiscNER		
	P	R	F1	P	R	F1	P	R	F1
Key Findings	0.7684	0.7772	0.7728	0.7260	0.6794	0.7020	0.7860	0.7800	**0.7832**
Normal Findings	0.5641	0.5641	0.5641	0.8071	0.7792	**0.7929**	0.7284	0.7867	0.7564
**Overall**	0.7453	0.7529	0.7491	0.7981	0.7678	**0.7827**	0.7786	0.7811	0.7799

The highest F1-scores are highlighted in bold.

We compare both models with DiscHPO and observe a notable improvement over the baseline, with approximately a 3% increase. When comparing our two models with each other, we notice that the overall performance is relatively comparable. However, at the per-class level, TriG-NER is better at detecting normal findings, while DocDiscNER shows superior performance in identifying key findings. Overall, TriG-NER achieves higher overall precision and F1 score, indicating the effectiveness of using TriG-NER on sentence-level datasets.

In Table 5, we report the NER results on the Occup-Sub, including per-class performance for each of the six entity types. Our models’ results are comparable to the span-based baseline, with DocDiscNER achieving a 1% increase in F1 score. This improvement can be attributed to the utilisation of the full functionality of DocDiscNER, including processing document-level inputs. In terms of per-class performance, DocDiscNER demonstrates a notable improvement over the baseline, with an F1 gain exceeding 12% for the “Activity” entity type. This improvement likely stems from the relatively high proportion of discontinuous entities in this category (more than 20%), as shown in Table 2.

**Table 5:** NER results of Occup-Sub on the test set, including both overall and per-class scores.

Model	Baseline (Span-based)			TriG-NER			DocDiscNER		
	P	R	F1	P	R	F1	P	R	F1
Exposure Measured	0.8500	0.8800	0.8600	0.8675	0.8724	**0.8699**	0.8473	0.8794	0.8630
Sample Type	0.8300	0.8700	**0.8500**	0.8378	0.8611	0.8493	0.8060	0.7500	0.7770
Industry/Workplace	0.7600	0.7000	0.7300	0.7239	0.7604	0.7414	0.7464	0.7574	**0.7518**
Occupation/Job Title	0.9000	0.8400	0.8700	0.8850	0.8556	**0.8701**	0.8810	0.8268	0.8530
OH Device	0.7400	0.7400	0.7400	0.7143	0.7197	0.7170	0.7872	0.8409	**0.8132**
Job Task/Activity	0.5900	0.6700	0.6300	0.7143	0.7282	0.7211	0.8256	0.6893	**0.7513**
**Overall**	0.8100	0.8100	0.8100	0.8119	0.8225	0.8172	0.8201	0.8243	**0.8222**

The highest F1-scores are highlighted in bold. Baseline scores were originally reported to two decimal places; trailing zeros have been appended for consistency.

### 4.2 Sentence-level ablation for DocDiscNER

A key methodological difference between the two models is their input granularity: TriG-NER operates on sentence-level inputs, whereas DocDiscNER processes document-level context through its semantic chunking mechanism. This raises a valid concern regarding whether DocDiscNER’s performance gains on Occup-Sub are attributable to its architecture or to the richer contextual signal afforded by longer inputs. To isolate this factor, we conduct an ablation experiment in which DocDiscNER is restricted to sentence-level inputs on the Occup-Sub dataset, matching the input level used by TriG-NER. We refer to this configuration as DocDiscNER (Sent.) throughout this section.

Table 6 presents the per-class and overall NER results for TriG-NER, DocDiscNER (document-level), and DocDiscNER (Sent.) on the Occup-Sub test set.

**Table 6:** NER results of Occup-Sub on the test set, including both overall and per-class scores for TriG-NER, DocDiscNER (document-level), and DocDiscNER restricted to sentence-level inputs (Sent.).

Model	TriG-NER			DocDiscNER			DocDiscNER (Sent.)		
	P	R	F1	P	R	F1	P	R	F1
Exposure Measured	0.8675	0.8724	0.8699	0.8473	0.8794	0.8630	0.8197	0.9388	**0.8752**
Sample Type	0.8378	0.8611	**0.8493**	0.8060	0.7500	0.7770	0.7654	0.8857	0.8212
Industry/Workplace	0.7239	0.7604	0.7414	0.7464	0.7574	**0.7518**	0.6725	0.8292	0.7427
Occupation/Job Title	0.8850	0.8556	0.8701	0.8810	0.8268	0.8530	0.8281	0.9191	**0.8712**
OH Device	0.7143	0.7197	0.7170	0.7872	0.8409	**0.8132**	0.6207	0.8438	0.7152
Job Task/Activity	0.7143	0.7282	0.7211	0.8256	0.6893	**0.7513**	0.5429	0.7755	0.6387
**Overall**	0.8119	0.8225	0.8172	0.8201	0.8243	**0.8222**	0.7444	0.8915	0.8113

The highest F1-scores are highlighted in bold.

Restricting DocDiscNER to sentence-level inputs leads to a clear drop in overall performance. DocDiscNER (Sent.) achieves an F1 of 0.8113, lower than both document-level DocDiscNER (0.8222) and TriG-NER (0.8172). Although it attains the highest recall (0.8915), this is accompanied by a substantial reduction in precision (0.7444).

We noticed that over-extraction was most frequent for the Industry/Workplace and Job Task/Activity entity types, which show the largest precision drops. In these cases, the model often predicts contextually plausible but non-annotated spans, such as general geographic names or broad activity phrases. We also observe more span boundary errors, where extra modifiers are included in the predicted mention (e.g., “background atmospheric aerosol” instead of “atmospheric aerosol”)

A third observation is the emergence of nested extraction behaviour, where the model extracts both a full span and individual tokens or sub-spans within it as separate entities. This pattern, also observed in TriG-NER’s predictions, suggests that sentence-level inputs may encourage models to produce overlapping predictions.

Despite this decline, DocDiscNER (Sent.) achieves the highest F1 for *Exposure Measured* (0.8752) and Occupation/Job Title (0.8712). These classes appear to rely more on distinctive lexical cues and are therefore less dependent on wider context.

### 4.3 Performance patterns in all NEs

In this section, we conduct a deeper span-level analysis of both models’ performance, identifying areas where they excel and struggle across different entity spans and dataset domains.

#### 4.3.1 Input length

**Figure 3:**
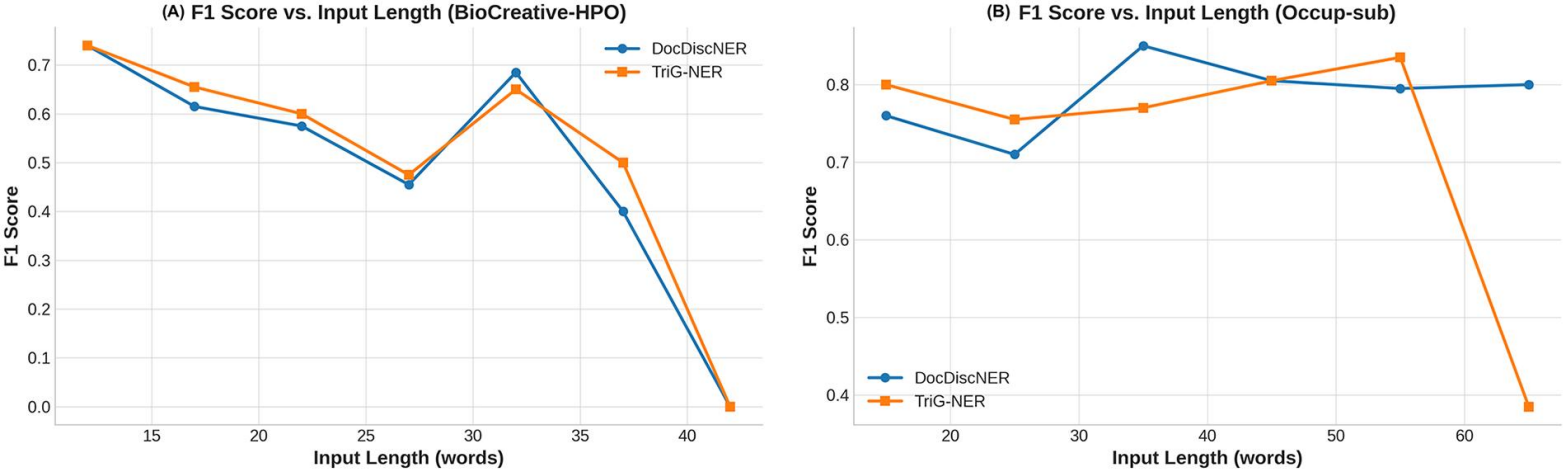
F1 scores across input lengths for both datasets. **(A)** BioCreative-HPO. **(B)** Occup-Exp.

In BioCreative-HPO (Figure 3A), both models achieve their highest performance on short sentences (∼12 words). Performance declines slightly as sentence length increases to around 27 words, then rises again near 32 words, before dropping for longer sentences.

In contrast, the Occup-sub dataset (Figure 3B) shows greater stability across most lengths. DocDiscNER peaks at ∼35 words and remains stable, whereas TriG-NER drops in longer sentences. The discrepancy between two models in longer sentences may be partly due to DocDiscNER’s predictions being made on the document-level version of Occup-sub rather than sentence-level, potentially boosting per-sentence performance relative to TriG-NER.

#### 4.3.2 Entity density

**Figure 4:**
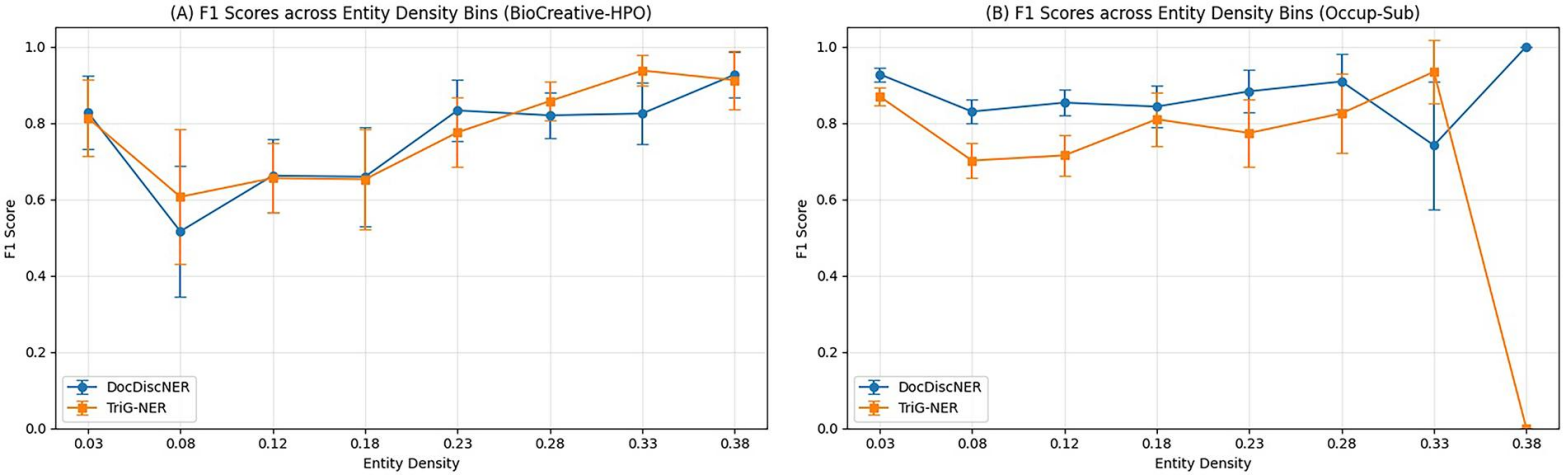
F1 scores across entity density bins. **(A)** BioCreative-HPO validation set. **(B)** Occup-Sub test set. Error bars indicate 95% confidence intervals computed from sentence-level F1 scores within each bin.

Figure 4 shows that model performance varies with entity density in both datasets, although the pattern differs across corpora. overall, we observe a consistent performance in both datasets where it improves as entity density increases. In BioCreative-HPO, TriG-NER achieves slightly higher mean F1 scores than DocDiscNER, suggesting an advantage in handling denser sentence-level inputs. In Occup-Sub, DocDiscNER shows stronger and more stable performance across low-to-moderate density bins, whereas TriG-NER outperforms in high-density scenarios. The extreme values observed in the final density bin for both datasets are based on a small number of instances.

#### 4.3.3 Out of vocabulary density

**Figure 5:**
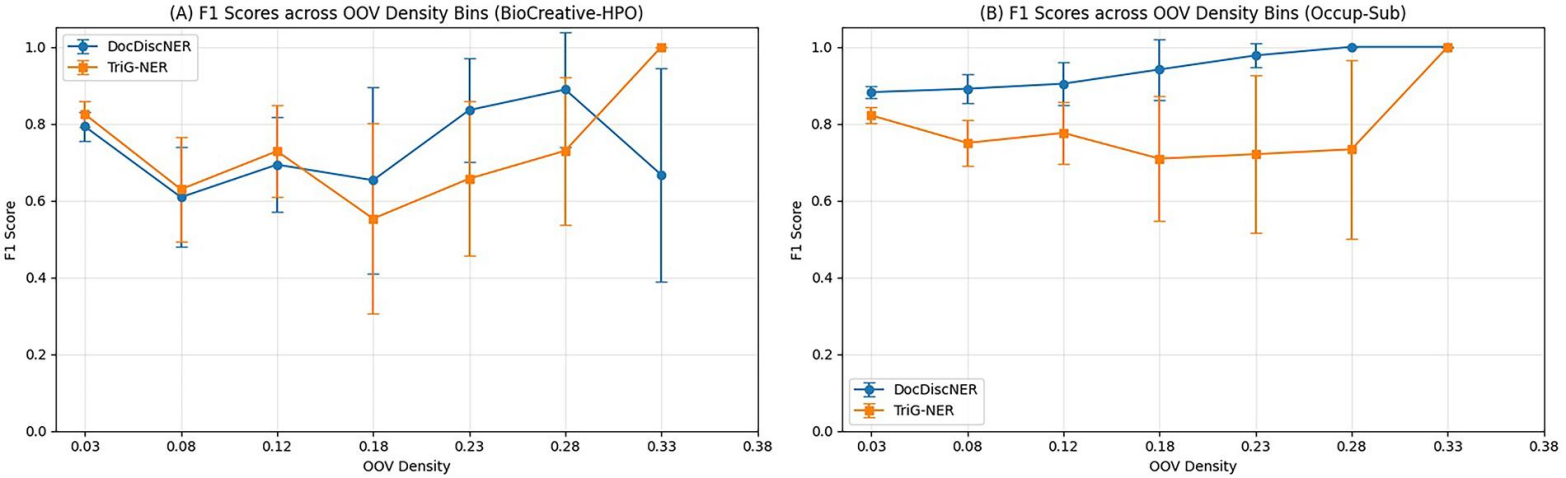
F1 scores across OOV density bins. **(A)** BioCreative-HPO validation set. **(B)** Occup-Sub test set. Error bars indicate 95% confidence intervals computed from sentence-level F1 scores within each bin.

Figure 5 compares the performance of DocDiscNER and TriG-NER across different OOV density bins. For BioCreative-HPO, both models are affected by increasing OOV density, with performance declining from the lowest-density bins. DocDiscNER generally shows stronger robustness in the middle and moderately high OOV bins, where it achieves higher mean F1 scores than TriG-NER. By contrast, TriG-NER exhibits greater variability, with wider confidence intervals and a marked drop in performance at some intermediate density levels. For the Occup-Sub dataset, DocDiscNER remains resilient across increasing OOV density levels, maintaining consistently high F1 scores even when a larger proportion of words are unseen during training. In contrast, TriG-NER shows lower and more variable performance across most OOV bins, indicating greater sensitivity to unfamiliar vocabulary.

### 4.4 DiscNER results

In Table 7, we specifically report the results of discontinuous mentions across both datasets.

**Table 7:** DiscNER results for the BioCreative-HPO dataset on the validation set and the Occup-Sub dataset on the test set.

Dataset	Entity type	TriG-NER			DocDiscNER		
		P	R	F1	P	R	F1
BioCreative-HPO	Key findings	0.7895∖0.6667	0.625∖0.5882	0.6977∖0.625	0.7325∖0.4185	0.6319∖0.625	0.6785∖0.5219
	Normal findings	0.7258∖0.6897	0.6429∖0.5063	0.6818∖0.5839	0.7309∖0.5	0.7037∖0.6842	0.7170∖0.5778
	**Overall**	0.7466∖0.4849	0.6416∖0.5208	0.6873∖0.5917	0.7322∖0.4443	0.6411∖0.6364	0.6837∖0.5468
Occup-Sub	Substance Measured	0.7755∖0.5625	0.7451∖0.4737	0.76∖0.5143	0.8133∖0.5217	0.8026∖0.6316	0.8079∖0.5714
	Sample Type	0.7273∖0.7778	0.6154∖0.5833	0.6667∖0.6667	0.8889∖1.00	0.5333∖0.5833	0.6667∖0.7368
	Industry/Workplace	1.00∖0.50	0.8750∖0.75	93.33∖0.60	0.6667∖0.00	0.6667∖0.00	0.6667∖0.00
	Occupation/Job Title	0.9286∖0.50	0.7647∖0.5556	0.8387∖0.5263	1.00∖1.00	0.8571∖0.7500	0.9231∖0.8571
	OH Device	0.20∖0.00	0.20∖0.00	0.20∖0.00	0.875∖1.00	1.00∖1.00	0.9333∖1.00
	Job Task/Activity	0.913∖0.7778	0.60∖0.35	0.7241∖0.4828	0.8519∖0.7857	0.6389∖0.55	0.7302∖0.6471
	**Overall**	0.7987∖0.6078	0.7384∖0.4559	0.7674∖0.521	0.8233∖0.7018	0.7457∖0.597	0.7866∖0.6452

Scores are separated by “∖”, where the first value represents performance on sentences containing discontinuous mentions, and the second denotes results for DiscNEs only.

On BioCreative-HPO, both models perform comparably in detecting DiscNEs, with TriG-NER showing better performance overall, particularly for detecting discontinuous mentions relative to continuous ones within the same sentence. At the per-class level, TriG-NER outperforms DocDiscNER in detecting discontinuous key findings, achieving approximately 2% higher F1 on sentences containing DiscNEs and around 10% higher F1 when evaluating DiscNEs only. Overall, this highlights TriG-NER’s stronger focus and capability in extracting discontinuous entities over their continuous counterparts within the same entity type.

For the Occup-Sub dataset, we observe that DocDiscNER achieves superior overall DiscNE performance across both evaluation metrics. In the per-class DiscNE results for the Occup-Sub dataset, DocDiscNER outperforms TriG-NER in detecting DiscNEs across most categories. This suggests that TriG-NER performs best in datasets with fewer entity types, while DocDiscNER is better suited to handling multiple entity categories.

### 4.5 Performance patterns in DiscNEs

Here, we further explore patterns in DiscNE performance, critically compare the strengths and weaknesses of both models, and discuss potential enhancements for discontinuous NER.

#### 4.5.1 Interval and span length

**Figure 6:**
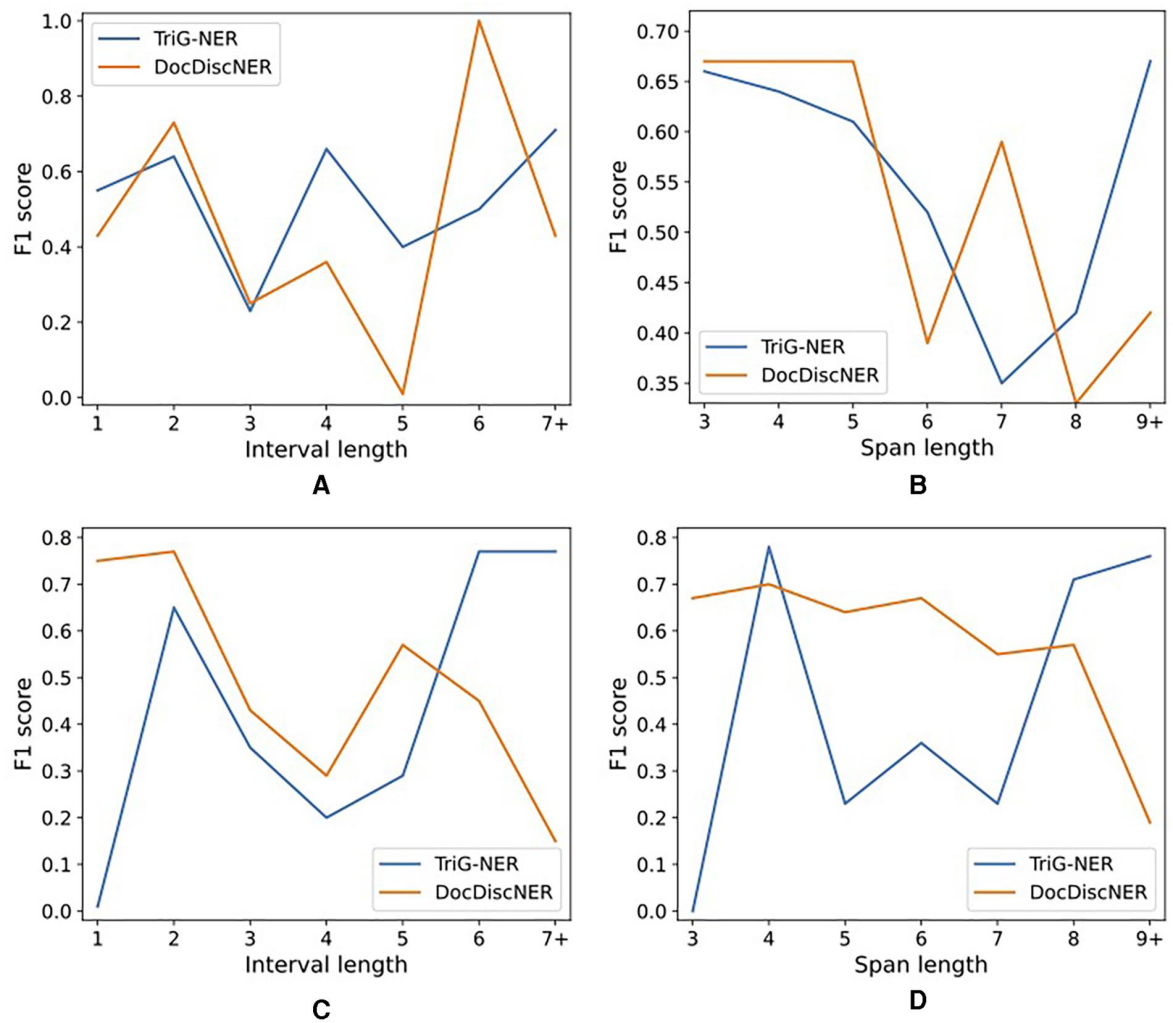
F1-score comparison of span length and interval length effects on discontinuous mentions. **(A)** BioCreative-HPO interval length. **(B)** BioCreative-HPO span length. **(C)** Occup-Sub interval length. **(D)** Occup-Sub span length.

In Figures 6A–D, we compare the performance of both models in detecting DiscNEs by analysing their results across different interval and span lengths.

For interval length, both models show a relatively similar performance when detecting DiscNEs across different interval sizes. However, some variations in performance are also observed. For instance, in the BioCreative-HPO dataset, TriG-NER outperforms across most interval lengths, except at interval length 6, where the number of instances is minimal (n=8). In the Occup-Sub dataset, DocDiscNER achieves higher performance up to interval length 5, after which its accuracy declines while TriG-NER continues to improve. This indicates that TriG-NER is more effective at modeling long-range dependencies, allowing it to better capture DiscNEs with larger numbers of intervening tokens. The sharp divergence observed at interval length 1 in the Occup-Sub dataset is not considered meaningful due to the very limited number of instances (n=4).

In terms of span length (Figures 6B,D), the performance patterns show less consistency across the two datasets. DocDiscNER performs better on shorter spans, while TriG-NER is more robust in handling spans longer than 9 tokens. However, a discrepancy occurs at a span length of 7, where DocDiscNER outperforms TriG-NER, despite the latter’s noticed overall strength in longer spans.

#### 4.5.2 Discontinuous structures

**Figure 7:**
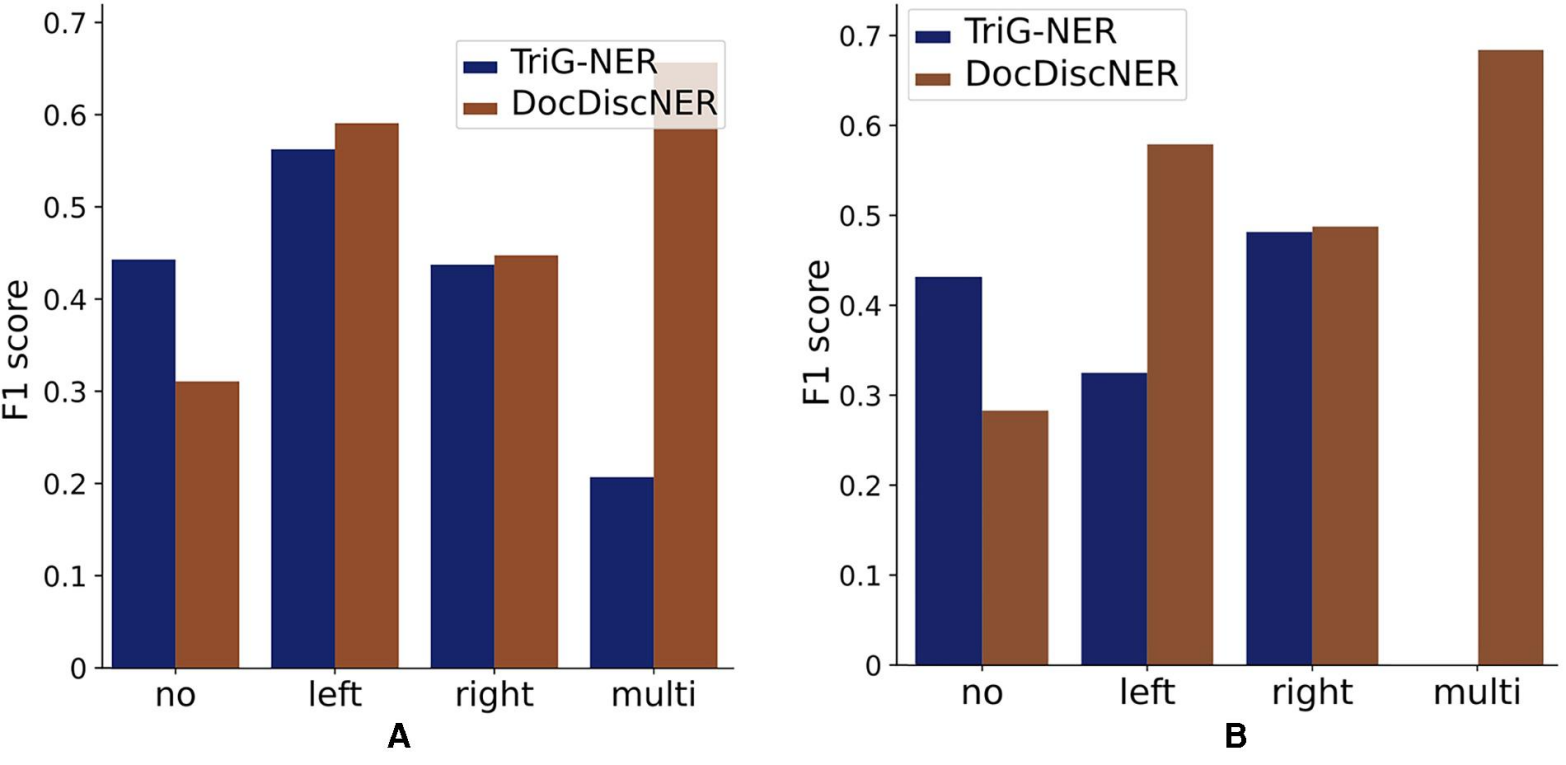
F1-Score comparison of different discontinuous structures. **(A)** BioCreative-HPO. **(B)** Occup-Sub.

As shown in Figures 7A,B, TriG-NER is more effective at extracting non-overlapping DiscNEs, outperforming DocDiscNER, which struggles with these cases, particularly in the Occup-Sub dataset, where non-overlapping DiscNEs outnumber overlapping ones (47 vs. 21 instances). For left and right overlapping mentions, both models demonstrate comparable performance, with DocDiscNER showing a slight advantage. This can be attributed to its CER component, which helps resolve shared heads from coordination expressions. However, TriG-NER also performs competitively, likely due to its tagging scheme, which has proven effective in identifying overlapping segments in previous studies (Mac, TOE, W2NER). When it comes to multiple overlapping heads, which are rare in both datasets (10 instances in BioCreative-HPO and only 2 in Occup-Exp), DocDiscNER is notably more effective. It successfully extracts 7 out of 10 instances in BioCreative-HPO and 1 out of 2 in Occup-Sub, while TriG-NER captures only 2 out of 10 in BioCreative-HPO and none in Occup-Sub.

### 4.6 Normalisation results

As reported in Table 8, the performance of several LLMs as normalisers when the gold standard entity spans were provided. This setup reflects a best-case scenario, where the normaliser is not affected by upstream extraction errors, and thus represents the upper-bound performance.

**Table 8:** Performance of various LLMs as normalisers when provided with gold standard spans.

Model	TP	FN	F1
Sentence-Transformer (roberta-large)	603	131	0.900
Llama-2-13b-hf	601	133	0.900
Llama-3.1-8B	580	154	0.882
*Instruction-tuning*			
Llama-3-8B-Instruct	617	117	**0.9134**
Llama-3.1-8B-Instruct	608	126	0.906
Mistral-7B-Instruct	505	229	0.815

The highest F1-scores are highlighted in bold.

Among the models evaluated, Llama-3-8B-Instruct achieved the highest F1 score of 0.9134, surpassing all other models. This includes both larger-scale base models such as Llama-2-13B-hf (F1 = 0.90) and sentence-transformer baseline (F1 = 0.90). The newer version instruction-tuned Llama3.1-8B-Instruct also performed competitively with F1 scores of 0.906, reflecting the advantage of instruction tuning, with instruction-tuned model outperforming its base counterpart in the normalisation task.

Moving from using gold standard spans to an end-to-end scenario, Table 9 presents normalisation results based on predicted spans, evaluated using the CodaLab evaluation platform on the BioCreative-HPO test set. We report results for three sub-tasks: normalisation, exact extraction and normalisation, and partial extraction and normalisation. These metrics are explained in detail in the BioCreative VIII overview paper [5].

**Table 9:** Normalised BioCreative-HPO test results using the CodaLab evaluation platform.

Model	Normalisation			Exact Extraction + Norm.			Partial Extraction + Norm.		
	P	R	F1	P	R	F1	P	R	F1
PhenoTagger	0.587	0.687	0.633	–	–	–	0.586	0.685	0.632
**PhenoID**	0.736	0.699	0.717	–	–	–	0.735	0.696	0.715
**DiscHPO**	0.7179	0.7281	0.7229	0.6899	0.6367	0.6623	0.7172	0.7258	0.7214
*Our models*									
**DocDiscNER**	0.7257	0.7552	0.7402	0.6439	0.6971	0.6697	0.7243	0.7496	0.7367
**TriG-NER**	0.7488	0.7383	**0.7433**	0.6768	0.6686	**0.6724**	0.7488	0.7383	**0.7433**

The highest F1-scores are highlighted in bold.

In the normalisation-only setting, both models—DocDiscNER and TriG-NER—achieved competitive performance. TriG-NER achieved the highest F1 score of 0.743, closely followed by DocDiscNER with 0.74. Compared to existing baselines such as DiscHPO (F1 = 0.723) and PhenoID (F1 = 0.717), both of our models demonstrate clear improvements in mapping entity mentions to HPO terms, showing their robustness even when relying on predicted NEs rather than gold standard spans.

For the exact extraction and normalisation task, performance decreased across all models due to the added challenge of requiring precise span boundaries. However, TriG-NER maintained a strong lead with an F1 score of 0.677, slightly ahead of DocDiscNER at 0.670, both outperforming the DiscHPO baseline.

The partial extraction and normalisation task, which is slightly more relaxed regarding the span boundaries, shows similar trends. TriG-NER again had the best performance (F1 = 0.7433), indicating that it is not negatively affected by minor span mismatches during extraction while still producing the correct normalisation outputs.

### 4.7 Statistical significance

To assess whether the observed performance differences were statistically reliable, we conducted paired bootstrap resampling [43] over model predictions for each dataset. In each iteration, instances were resampled with replacement, and corpus-level micro-F1 was recomputed from the aggregated TP, FP, and FN counts. All tests used B=10,000 bootstrap samples and report two-sided p-values.

For BioCreative-HPO, resampling was evaluated across the three normalisation settings defined by the shared task. Comparing each model against the DiscHPO baseline, neither DocDiscNER nor TriG-NER demonstrated statistically significant improvements across the normalisation-only and overlapping extraction and normalisation settings. TriG-NER was the only model to achieve a statistically significant gain over the baseline, exclusively for exact extraction and normalisation (p=0.013), while DocDiscNER did not reach significance on any setting (p>0.05). In the direct comparison between DocDiscNER and TriG-NER, TriG-NER outperformed DocDiscNER on exact extraction and normalisation (p=0.035) and overlapping extraction and normalisation (p=0.043), while the difference for normalisation only did not reach significance (p=0.052).

For Occup-Sub, it should be noted that testing against the span-based baseline was not conducted for this dataset, as its predictions were not available to us; significance testing was therefore restricted to the direct comparison between DocDiscNER and TriG-NER. The overall difference between DocDiscNER and TriG-NER was significant (p<0.001). At the per-class level, DocDiscNER achieved significantly higher F1 scores for three of the six entity types: *Industry/Workplace* (p<0.001), *Job Task/Activity* (p<0.001), and *OH Device* (p<0.001).

### 4.8 Computational cost analysis

We evaluated the computational efficiency of DocDiscNER and TriG-NER under a consistent experimental setup using the BioCreative-HPO and Occup-Sub datasets. Table 10 summarises key metrics including total training time (in seconds), average time per epoch, number of trainable parameters, GPU and system RAM consumption, and disk usage.

**Table 10:** Efficiency comparison of TriG-NER and DocDiscNER. *Training Time* refers to the total time taken to train the model across all epochs, with values reported in both *hh:mm:ss* and seconds.

Attribute	TriG-NER		DocDiscNER	
	Occup-Sub	BioCreative-HPO	Occup-Sub	BioCreative-HPO
Training Time (s)	03:33:22 (12802.13s)	00:37:29 (2249.27s)	03:58:30 (14310.3s)	00:54:33 (3273.35s)
Trainable Parameters	245.68M	245.68M	369M	369M
Base Model	BioBERT v1.2	BioBERT v1.2	Mistral-7B	BioMistral-7B
GPU RAM Used	18.8 GB	13.7 GB	22.2 GB	18.0 GB

TriG-NER achieved its best results after 46 epochs, whereas DocDiscNER was trained for 20 epochs. Despite its fewer epochs, DocDiscNER consumed more training time overall, especially for the HPO dataset. Specifically, TriG-NER required 31% less total training time on HPO (2249s vs. 3273s), and approximately 10.5% less time on the Occup-Sub dataset (12802s vs. 14310s).

In terms of memory efficiency, DocDiscNER consumed approximately 4 GB more GPU RAM compared to TriG-NER across both datasets. This aligns with its larger model size (i.e., 369M trainable parameters vs. 245M in TriG-NER). Despite its higher memory footprint, DocDiscNER remained within the limits of the L4 GPU’s 24 GB capacity.

### 4.9 Few-shot performance analysis of GPT-4.1

As shown in Table 11, for the Occup-Sub dataset, performance metrics improve consistently with the number of shots, indicating that GPT-4.1 benefits from additional context in this domain. For the NER results on the BioCreative-HPO validation set, the 4-shot configuration achieves the highest performance. A slight decline in F1 score is observed in the 6-shot setting. The NEN results on the test set remain consistently low across all settings, with only minor improvement at 6 shots.

**Table 11:** NER performance of GPT-4.1 in a few-shot setting (2, 4, and 6 shots) on the Occup-Sub test set and BioCreative-HPO validation set, alongside NEN results on the BioCreative-HPO test set.

Dataset	2-Shots			4-Shots			6-Shots		
	P	R	F1	P	R	F1	P	R	F1
Occup-Sub	0.298	0.448	0.358	0.297	0.522	0.378	0.399	0.646	0.494
BioCreative-HPO (Valid-NER)	0.507	0.576	0.539	0.523	0.602	0.559	0.500	0.582	0.538
BioCreative-HPO (Test-NEN)	0.288	0.315	0.301	0.283	0.319	0.299	0.285	0.322	0.303

## 5 Discussion

### 5.1 Comparative model analysis

Both models demonstrate complementary strengths across different entity types and structural complexities. In terms of input length, short to medium sentences yield higher accuracy for both models, while very long sentences substantially degrade performance. An exception is observed with DocDiscNER on the Occup-Sub dataset, where performance remains steady even at higher sentence lengths, likely due to its document-level processing approach. Regarding entity density, higher densities generally lead to better F1 performance, particularly for TriG-NER, suggesting its resilience in handling multiple named entities within a single input sentence. For OOV density, the wider error bars for TriG-NER at higher OOV densities suggest reduced stability under lexically challenging vocabulary. However, DocDiscNER shows stronger resilience in handling unseen vocabulary than TriG-NER, demonstrating a marginal advantage in moderate to high OOV density bins.

With respect to discontinuous entity structures, TriG-NER demonstrates a clear advantage in handling DiscNEs with long interval lengths and extended spans, reflecting its ability to model long-range dependencies effectively through word-pair similarities and dissimilarities within a grid structure. In contrast, DocDiscNER shows stronger performance on shorter spans and benefits from its CER component, which provides an advantage in handling overlapping entities, including those with multiple shared heads. Specifically, DocDiscNER successfully extracts 7 out of 10 multiple-overlap instances in BioCreative-HPO, compared to only 2 captured by TriG-NER. However, DocDiscNER struggles with non-overlapping DiscNEs, particularly in the Occup-Sub dataset where non-overlapping instances outnumber overlapping ones (47 vs. 21 instances).

Overall, the choice between the two models should be guided by the structural characteristics of the target dataset: TriG-NER is better suited for datasets with predominantly non-overlapping and long-range discontinuous entities, while DocDiscNER is preferable when overlapping and multiple-head structures are more frequent.

### 5.2 Computational trade-offs

While both models are compatible with cost-effective hardware such as the NVIDIA L4, TriG-NER demonstrates greater overall efficiency, with shorter training times and lower memory usage. TriG-NER required 31% less total training time on the BioCreative-HPO dataset (2249s vs. 3273s) and approximately 10.5% less time on the Occup-Sub dataset (12802s vs. 14310s). The smaller time gap on Occup-Sub is likely because DocDiscNER processes data at the document level, which inherently increases per-epoch processing time relative to TriG-NER’s sentence-level approach. In terms of memory, DocDiscNER consumed approximately 4 GB more GPU RAM across both datasets, consistent with its larger parameter count (369M vs. 245M). These trade-offs suggest that TriG-NER is particularly suitable for scenarios where resource efficiency is a key constraint, whereas DocDiscNER’s higher resource demands may be justified in settings that require stronger handling of complex overlapping discontinuous structures.

### 5.3 Limitations of few-shot prompting

The few-shot results reveal important limitations of GPT-4.1 under prompt-only conditions for biomedical and occupational NER. While performance improves consistently with shot count on the Occup-Sub dataset, the BioCreative-HPO validation results show a decline at the 6-shot setting, suggesting that introducing DiscNE examples into the prompt context may introduce noise or cause the model to overfit to discontinuous patterns at the expense of continuous span recognition. More critically, NEN performance on the BioCreative-HPO test set remains consistently low across all configurations, with only marginal improvement at 6 shots. This highlights the inherent difficulty of entity normalisation under few-shot conditions and suggests that full fine-tuning using the HPO dictionary is likely necessary, as few-shot prompting alone appears insufficient for this task. These results demonstrates a gap between general-purpose LLMs and task-specific fine-tuned models in specialised domains.

### 5.4 Qualitative error analysis

Slight performance disparity has been identified by the previous quantitative analysis. Here, we qualitatively analyse both continuous and discontinuous error patterns by manually reviewing examples and categorising error types. We identify common challenges such as boundary mismatches, incorrect extractions, and misclassification (wrong entity type), which impact overall performance.

#### 5.4.1 Boundary mismatches

This type of error occurs when the extracted NE is either partially extracted (missing NE tokens) or involves over-extended extractions (including extra non-NE tokens). We observed several behaviours in the models when predicting incorrect boundaries. In this analysis, we focus on the BioCreative-HPO validation set, as it provides normalised spans as part of a downstream application, making the study of boundary extraction patterns particularly relevant.

Partial extractions occurred in 15 cases for DocDiscNER and 11 for TriG-NER, where the models occasionally excluded tokens they deemed less relevant to the overall meaning of the NE. For example, both models omitted “mild” from the gold annotation “EYES—mild up-slant”, and TriG-NER excluded “appearing” from “EARS—Large appearing.” However, these omissions did not affect correct normalisation. In contrast, both models sometimes excluded critical tokens that are essential for accurate normalisation, such as body parts (e.g., nose, forehead) or laterality indicators (e.g., bilateral 2nd toe, left hand). For instance, both models extracted “Nevus flammeus” instead of the full gold span “Nevus flammeus—forehead”. Similarly, TriG-NER extracted “Large vein” from “Large vein on—forehead,” where mentioning the forehead is vital for correct normalisation. In this case, the missing token is discontinuous, which posed additional challenges for the models and led to normalisation failures.

We observed cases of extended extractions where the models occasionally included additional tokens they considered relevant, particularly body parts, even when these were not part of the gold annotation. This was observed more often in TriG-NER (19 cases) than in DocDiscNER (15 cases). For example, TriG-NER extracted “FACE—edema” instead of the gold annotation “edema”, where including “FACE” seemed more useful for downstream applications. In DocDiscNER, extended extractions were more commonly related to non-overlapping DiscNEs. For instance, it extracted “feet appear short” instead of the gold span “feet—short”. This challenge has been discussed earlier in Figure 7A, where DocDiscNER struggled to accurately extract non-overlapping DiscNEs.

Beyond partial and extended extractions, we also observed a consistent behaviour in TriG-NER, occurring in 25 instances, where the model extracted both a complete span and an extra nested span within it. For example, TriG-NER extracted both “hypertrichosis of forehead” and “hypertrichosis”, with the latter matching the gold standard and being correctly normalised. While this behaviour introduced additional false positives, it also reduced false negatives by providing a correct alternative, ultimately improving detection performance. This behaviour was not observed in DocDiscNER.

#### 5.4.2 Incorrect extraction

This category refers to cases where the models extracted spans that do not match any gold-standard annotations, even partially. In other words, these are false positives where the extracted tokens are completely unrelated to the annotated entities. We analysed the false-positive cases produced by both models and observed several notable patterns. One key observation is that both models share many of the incorrect extraction errors. These errors often arise because incorrectly predicted token spans contain features that closely resemble true entities in the training data. For example, models often confuse pure medical findings with side observations or physical signs that may suggest a finding but are not findings themselves. For example, both models incorrectly extracted “microcystic areas” from the sentence “microcystic areas on tongue” and also extracted “stridor”, which is a breathing sound that might indicate an underlying finding but is not a finding on its own. In addition, the models sometimes predicted findings that are temporary or age-specific. For example, “Overriding of sutures” and “Anterior fontanelle open” are normal physiological processes shortly after birth and typically resolve within the first few days. However, these same findings could be considered abnormal at older ages. This suggests that providing contextual information, such as the age of the patient, could help the models better distinguish between normal and abnormal findings. Finally, we identified a small set of outlier cases (three instances) where the models misclassified completely normal findings as abnormal, regardless of age, gender, or other human characteristics. Examples include “single uvula”, “palpebral fissures open”, and “Y-shaped gluteal cleft”. Upon reviewing the training data, we found similar examples but annotated differently, such as “bifid uvula”, “downslant palpebral fissures”, and “asymmetric gluteal cleft”. This indicates that the models lacked sufficient medical knowledge to correctly distinguish between normal and abnormal cases for these specific findings.

#### 5.4.3 Misclassification

This refers to cases where the entity type is incorrect; we focus primarily on the Occup-Sub dataset, as it contains six entity types and is therefore more prone to misclassification errors. For TriG-NER, the most frequently misclassified entity type is “Place”, with the model incorrectly labelling six different NEs. These misclassifications often involve confusion between “Place” and “Activity” classes. For example, the NE: “table grape production” was misclassified as an “Activity” when it should belong to the “Place” class. Similarly, both models classified “manual harvest operations” as an “Activity” when it should have been a “Place”. This suggests that distinguishing between entities ending in “operations” is particularly challenging, as their meanings resemble actions, even though they actually denote workplaces or industrial areas. On the other hand, DocDiscNER misclassified four NEs of the “Title” class, despite their relatively straightforward syntactic structure. For instance, “Bus garage mechanics”, “workers in quarries”, and “workers in foundries” were misclassified as Place. A common pattern among these misclassifications is the presence of location-related terms within the span (e.g., garage, quarries, foundries), which likely misled DocDiscNER into categorising them as “Place” entities. In contrast, TriG-NER correctly classified these spans but demonstrated a different behaviour: it extracted the embedded location names (“garage”, “quarries”, “foundries”) as additional nested entities. This tendency to enumerate nested spans, as noted in Section 5.4.2, suggests that TriG-NER performs particularly well on datasets where nested entities are explicitly annotated.

## 6 Conclusion

This study presents a comprehensive comparative analysis of two state-of-the-art approaches—TriG-NER and DocDiscNER—for the recognition and linking of DiscNEs in healthcare, alongside the evaluation of GPT-4.1 in a few-shot setting. Using two healthcare datasets (Occup-Sub and BioCreative-HPO) we provide insights into the strengths and limitations of different approaches for addressing this challenging task. Our findings demonstrate key characteristics of the models: TriG-NER achieves better performance on sentence-level datasets with fewer entity types, while DocDiscNER is more effective in handling multiple entity categories by leveraging a broader document-level context. An additional strength of TriG-NER lies in its capacity to process non-overlapping DiscNEs with extended spans and interval lengths, whereas DocDiscNER performs more effectively on complex overlapping structures and compact DiscNEs. In terms of computational efficiency, TriG-NER demonstrates clear advantages, requiring shorter training times and less GPU memory compared to DocDiscNER. While GPT-4.1 shows potential, its performance under few-shot conditions was limited, suggesting that full fine-tuning or adaptation strategies are needed to maximise its effectiveness in DiscNER tasks. Overall, this work demonstrates the importance of selecting domain-adapted models tailored to different levels of text granularity and specific entity structures challenges. By analysing performance patterns and computational trade-offs, our study contributes to advancing robust and efficient solutions for DiscNER, supporting downstream applications such as entity normalisation.

## Data Availability

The datasets used in this study are publicly available from the original sources: BioCreative-HPO [5] and Occup-Sub [24]. The dataset edits made in this study are provided in the article and/or Supplementary Material. Further enquiries can be directed to the corresponding author(s).
