# Supplementary material for: Recognition and linking of discontinuous named entities in healthcare: a comparative performance analysis
Source: Front Digit Health. 2026 Jun 9;8:1758921. doi: 10.3389/fdgth.2026.1758921 (PMC13288325; doi:10.3389/fdgth.2026.1758921)
Supplement: Supplementary file 1 [file Datasheet1.pdf]

frontiers

Supplementary Material

1 SUMMARY OF CORRECTED INCONSISTENT ANNOTATIONS

1.1 BioCreative-HPO Dataset

Table S1 summarises the seven inconsistencies identified in the BioCreative-HPO training and validation sets. The presented NEs follow the DocDiscNER format, for example: "KEYF: right cheek enlarged", where "KEYF" denotes the key finding entity type, and "right cheek enlarged" represents the annotated NE.

Observation ID	NEs before correction	NEs after correction	Correction
**Training Set**			
1f284729e22625ef54f	KEYF: right cheek enlarged right lip enlarged	KEYF: right cheek enlarged; KEYF: right lip enlarged	Split the NE into two NEs
318f62714250c099293	KEYF: rhizomelia)	KEYF: rhizomelia	Removed the ")"
f8515ae86ddf48e8b5d	KEYF: contractures of wrists; KEYF: contractures of wrists hands; KEYF: contractures of wrists, knees	KEYF: contractures of wrists; KEYF: contractures of hands; KEYF: contractures of knees	Removed the repetitive "wrist" since it is not part of the HPO concept
**Validation Set**			
49ca2e28bd866592d64	NORMF: NA; NORMF: NA	NORMF: No frontal bossing; NORMF: Normal shape of the head	Added a space between "bossing" and "Normal" in "bossingNormal"
4ffadcd13f74268fc0e	KEYF: Flat, nasal bridge	KEYF: Flat nasal bridge	Removed the comma after "flat"
b81b67f3c58ccb6feac	KEYF: Long toes and	KEYF: Long toes	Removed the extra "and"
d021a7a4f209bf9101f	KEYF: Widely separated, teeth	KEYF: Widely separated teeth	Removed the comma after "separated"

Table S1. The identified inconsistencies in the BioCreative-HPO dataset. For readability, only the first 19 characters of each 32-character Observation ID are shown

1.2 Occup-Sub Dataset

1.2.1 Merged sentences

Table S2 illustrates cases in which a named entity was incorrectly divided between two sentences. To correct this inconsistency, we merged any sentence pairs containing an entity that spanned across them. This preprocessing step was applied mainly for TriG-NER, given that the model operates at the sentence level.

Document ID	Sentence1 ID	Sentence2 ID	NE ID	NE text
Groves_et_al	S3276	S3277	A3844	Gastec Dosi No. 1 DL tubes
Groves_et_al	S3279	S3280	A3846	Draeger 500/a-D or Gastec Dosi No. 2 D tubes
Groves_et_al	S3283	S3284	A3848	Gastec Dosi No. 9 D tubes
Hewett_and_Bullock	S3577	S3578	A4215	locomotive trailing
Liukonen_et_al	S4080	S4081	A5043	pusher locomotives
Liukonen_et_al	S4080	S4081	A5044	helper locomotives
Liukonen_et_al	S4085	S4086	A5053	personal samples
Seshaidri_2003	S5010	S5011	A6228	37-mm diameter open-faced cassettes
Shih_et_al_2008	S5267	S5269	A6694	carbon organic
Shih_et_al_2008	S5267	S5268	A6695	carbon elemental
Shih_et_al_2008	S5350	S5351	A6791	car lanes, with tickets
Shih_et_al_2008	S5355	S5356	A6805	car lanes, with tickets
Shih_et_al_2008	S5358	S5359	A6809	car lanes, with tickets
hammond_1988	S5777	S5778	A7442	Total particles
hammond_1988	S5777	S5778	A7443	respirable particles
pratt_1997	S6158	S6159	A7903	mining operation
pratt_1997	S6181	S6182	A7931	Univ. of Minnesota/U.S. Bureau of Mines sampling device
roegner2002	S6289	S6290	A8054	gas-phase constituents
stanevich1997	S6503	S6504	A8271	Mine Safety AppliancesCo cassette
Healy_et_al_2014	S9114	S9115	A11759	grinding sandstone
Kim_et_al_2021	S9287	S9288	A11978	SKC Cat No. 225-2LF
Kim_et_al_2021	S9290	S9291	A11981	SKC Cat No. 225-01-02
Shih_et_al_2008	S11338	S11339	A14770	Part No. 456243
Shih_et_al_2008	S11339	S11341	A14772	Cat. No. 225-1

Table S2. List of the sentence pairs merged into a single sentence in the Occup-Sub dataset. The IDs correspond to the original sentences and named entity identifiers as provided in the JSON version of the dataset.

1.2.2 Corrected NEs

Table S3 presents a list of named entities in which whitespace was mistakenly omitted between a token and the entity during the original corpus annotation process. We have corrected these cases by inserting the missing spaces in their proper positions.

Sentence ID	NE ID	Inconsistency	Corrected NE
S1460	A1751	ESDPM	DPM
S1464	A1767	ESDPM	DPM
S1943	A2323	PM2.5and	PM2.5
S1957	A2348	bothP&D drivers	P&D drivers
S1971	A2386	andOC	OC
S2343	A3078	ormation of expansions for linking platforms	formation of expansions for linking platforms
S2445	A3133	ands enior management	senior management
S2526	A3194	soEC	EC
S2564	A3262	andOC	OC
S2754	A3404	sP&D/dockworkers	P&D/dockworkers
S2772	A3419	for long-haul drivers	long-haul drivers
S2869	A3463	toPM2.5	PM2.5
S2882	A3487	thatPM2.5	PM2.5
S2950	A3604	obtainPEM	PEM
S3113	A3762	SinceP&D trucks	P&D trucks
S3275	A3841	ong-term diffusive colorimetric gas detector tubes	long-term diffusive colorimetric gas detector tubes
S3577	A4204	locomotive ead	locomotive lead
S3742	A4434	OCEC carbon	OC carbon
S3742	A4435	OCEC carbon	EC carbon
S3909	A4721	PM10respectively	PM10
S4334	A5367	25x105particles	particles
S4681	A5765	ransport-industry environment	transport-industry environment
S5062	A6300	diesel freight termina	diesel freight terminal
S5124	A6385	toEC	EC
S5222	A6589	mechanicsf	mechanics
S5445	A7004	ofEC	EC
S6109	A7819	personalsamples	personal samples
S6836	A8714	mine porta	mine portal
S6806	A8773	haul coa	haul coal
S7019	A9012	truck bay7	truck bay
S7044	A9072	7-mm closed- face cassette	37-mm closed- face cassette
S7045	A9075	construction dus	construction dust
S8127	A10520	10-mm nylon sample	10-mm nylon sampler
S8421	A10751	core dri	core drill
S8501	A10857	models 1200	model 1200
S9475	A12258	logTD	TD
S9478	A12263	logRD	RD
S10245	A13317	respirable u	respirable u respirable quartz
S10380	A13502	respirable dust1	respirable dust
S10380	A13503	inhalable dust1	inhalable dust
S10648	A13927	s	crystalline
S10902	A14276	vessels apply protective coatings to	apply protective coatings to vessels
S10902	A14277	apply protective coatings to	apply protective coatings to large diameter piping
S11417	A14882	0 mm nylon cyclone	10 mm nylon cyclone
S11788	A15336	ground-work sub-contractor	ground-work sub-contractors
S12607	A16023	r quartz	respirable quartz
S12721	A16127	construction workers for	construction workers

Table S3. List of NEs with previously omitted white spaces between a token and the entity. The missing spaces have been restored by inserting them in their proper positions.

2 GPT-4.1 FEW-SHOT CONFIGURATIONS

In this section, we present the complete API prompts along with example inputs for each few-shot configuration across both datasets. For each dataset, the first subsection presents the head prompt, while the following subsections provide few-shot examples appended to the end of the prompt at each call.

2.1 BioCreative-HPO Dataset

2.1.1 Full BioCreative-HPO prompt:

```
SYSTEM_PROMPT = "You are an intelligent clinical Named Entity Recognition (
   NER) and Normalization (NEN) system. I will provide you the definition of
    the entities you need to extract and the ontology you need to map the
   extracted spans toto and the sentence from which you need to extract the
   entities and the output in given format with examples."
USER_PROMPT_1 = "Are you clear about your role?"
ASSISTANT_PROMPT_1 = "Sure, I'm ready to help you with your clinical NER and
    NEN tasks. Please provide me with the necessary information to get
   started."
PROMPT_TEMPLATE = (
"Entity Definition:\n"
"1. KEYF: Key clinical findings(Abnormal findings) , such as (low set ears)
   or (5th finger clinodactyly).\n"
"2. NORMF: Normal clinical findings E.g. (EYES: no up-slanting) or (normal
   head shape).\n"
"The Ontology to map extracted entities: The Human Phenotype Ontology, each
   span should be normalized to the closest unambiguous HPO ID such as \"HP
   :0000218\" for \"High arched palate\" and \"HP:0001009\" for \"no
   telangiectases\". \n"
"\n"
"Output Format for 1 example:\n"
"Spans: KEYF: Sparse eyebrow; NORMF: Normal lashes; NORMF: Normal lids\n"
"HPOs: HP:0045075; HP:0000499; HP:0000492\n"
"If no entities are presented in any categories keep it NA\n"
"\n"
"Examples:\n"
.
.
.
)
```

2.1.2 Two-shots exampels (DiscNEs not introduced)

```
"1. Sentence: EYES: Bruising over the eyelids, hyperteloric appearance,
   eyelid edema, slight down slant.\n"
"KEYF: eyelid edema; KEYF: EYES: down slant; KEYF: hyperteloric; KEYF:
   Bruising\n"
"HP:0100540; HP:0000494; HP:0000316; HP:0031364\n"
```

```
"\n"
"2. Sentence: GENERAL: Alert, active and well-nourished. Very ticklish.\n"
"NA\n"
"NA\n"
```

2.1.3 Four-shots with one example of DiscNEs (overlapping)

```
"1. Sentence: EYES: Bruising over the eyelids, hyperteloric appearance,
   eyelid edema, slight down slant.\n"
"KEYF: eyelid edema; KEYF: EYES: down slant; KEYF: hyperteloric; KEYF:
   Bruising\n"
"HP:0100540; HP:0000494; HP:0000316; HP:0031364\n"
"\n"
"2. Sentence: GENERAL: Alert, active and well-nourished. Very ticklish.\n"
"NA\n"
"NA\n"
"\n"
"3. Sentence: HANDS FEET: slight sandal gap, no polydactyly, very partial
   syndactyly of 23 toes, slight valgus deformity of great toe and 2nd toe.
   \n"
"KEYF: sandal gap; NORMF: no polydactyly; KEYF: syndactyly of 23 toes; KEYF:
    valgus deformity of great toe \n"
"HP:0001852; HP:0010442; HP:0004691; HP:0001822\n"
"\n"
"4. Sentence: MOUTH: Prominent and wide philtrum. Small cleft in upper gum
   line. High palate.\n"
"KEYF: Prominent philtrum; KEYF: wide philtrum; KEYF: Small cleft in upper
   gum line; KEYF: High palate\n"
"HP:0002002; HP:0000289; HP:0010289; HP:0000218\n"
```

2.1.4 Six-shots with two examples of DiscNEs (overlapping and non-overlapping)

```
"1. Sentence: EYES: Bruising over the eyelids, hyperteloric appearance,
   eyelid edema, slight down slant.\n"
"KEYF: eyelid edema; KEYF: EYES: down slant; KEYF: hyperteloric; KEYF:
   Bruising\n"
"HP:0100540; HP:0000494; HP:0000316; HP:0031364\n"
"\n"
"2. Sentence: GENERAL: Alert, active and well-nourished. Very ticklish.\n"
"NA\n"
"NA\n"
"\n"
"3. Sentence: HANDS FEET: slight sandal gap, no polydactyly, very partial
   syndactyly of 23 toes, slight valgus deformity of great toe and 2nd toe.\
   n"
```

```
"KEYF: sandal gap; NORMF: no polydactyly; KEYF: syndactyly of 23 toes; KEYF:
    valgus deformity of great toe. \n"
"HP:0001852; HP:0010442; HP:0004691; HP:0001822\n"
"\n"
"4. Sentence: MOUTH: Prominent and wide philtrum. Small cleft in upper gum
   line. High palate.\n"
"KEYF: Prominent philtrum; KEYF: wide philtrum; KEYF: Small cleft in upper
   gum line; KEYF: High palate\n"
"HP:0002002; HP:0000289; HP:0010289; HP:0000218\n"
"\n"
"5. Sentence: EYES: Edematous eyelids, appears wide-spaced with short
   palpebral fissures.\n"
"KEYF: Edematous eyelids; KEYF: short palpebral fissures; KEYF: EYES: wide-
   spaced\n"
"HP:0100540; HP:0012745; HP:0000316\n"
"\n"
"6. Sentence: NAILS HAIR SKIN: No penile freckling noted. Has nevus simplex.
    There is very slight redness of the left ear.\n"
"NORMF: No penile freckling; KEYF: nevus simplex; KEYF: redness\n"
"HP:0031447; HP:0001052; HP:0010783\n"
```

2.2 Occup-Sub Dataset

2.2.1 Full Occup-Sub prompt:

```
SYSTEM_PROMPT = "You are an intelligent clinical Named Entity Recognition (
   NER) system. I will provide you the definition of the entities you need
   to extract and the sentence from which you need to extract the entities
   and the output in given format with examples."
USER_PROMPT_1 = "Are you clear about your role?"
ASSISTANT_PROMPT_1 = "Sure, I'm ready to help you with your NER task. Please
    provide me with the necessary information to get started."
PROMPT_TEMPLATE = (
"Entity Definition:\n"
"1. PLACE: any description referring to an economic activity, such as (
   diesel factory) or (the construction of buildings).\n"
"2. TITLE: characterises a person or group of people in terms of their
   occupation, job title, position E.g. (electricians) or (carpenters).\n"
"3. ACTIVITY: Specific physical activities or actions that are carried out
   by workers as part of their daily working duties. E.g. (welding) or (
   concrete pouring).\n"
"4. SUBSTANCE: The name of a chemical or pollutant (a recognised exposure
   entity) that is measured or sampled. Such as (Elemental carbon) and (
   diesel exhaust).\n"
```

```
"5. DEVICE: The device, tool, apparatus, or sampling head used to measure
   levels of particulate and gaseous exposures by occupational hygienists in
    the workplace. E.g. (Higgins Dewell cyclones), (large cyclone separator)
   .\n"
"6. SAMPLE: Phrases denoting that collected samples of airborne substances,
   chemicals or pollutants represent personal exposures. E.g. (personal
   breathing zone sample), or (personal full-shift samples).\n"
"\n"
"Output Format example:\n"
"PLACE: immersed tunnel tubes; PLACE: ITT; PLACE: construction; ACTIVITY:
   curing of the concrete; TITLE: carpenters\n"
"If no entities are presented in any categories keep it NA\n"
"\n"
"Examples:\n"
.
.
.
)
```

2.2.2 Two-shots exampels (DiscNEs not introduced)

```
"Examples:\n"
"1. Sentence: Sampling of personal exposure to elemental carbon, nitrogen
   dioxide and inhalable dust was recorded.\n"
"Output: SAMPLE: Sampling of personal exposure; SUBSTANCE: elemental carbon;
    SUBSTANCE: nitrogen dioxide; SUBSTANCE: inhalable dust\n"
"\n"
"2. Sentence: Eligible subjects were identified from the company health-
   service registry according to the following entry criteria: 1) no history
    of respiratory disorder, including bronchitis or any other respiratory
   symptoms.\n"
"Output: NA\n"
```

2.2.3 Four-shots with one example of DiscNEs (overlapping)

```
"Examples:\n"
"1. Sentence: Sampling of personal exposure to elemental carbon, nitrogen
   dioxide and inhalable dust was recorded.\n"
"Output: SAMPLE: Sampling of personal exposure; SUBSTANCE: elemental carbon;
    SUBSTANCE: nitrogen dioxide; SUBSTANCE: inhalable dust\n"
"\n"
"2. Sentence: Eligible subjects were identified from the company health-
   service registry according to the following entry criteria: 1) no history
    of respiratory disorder, including bronchitis or any other respiratory
   symptoms.\n"
"Output: NA\n"
```

```
"\n"
"3. Sentence: The most important sources include diesel exhaust and rock
   dust, which are vortexed into the air by the traffic in the mine, and/ or
    dust drilling, loading and crushing of the rock/ore.\n"
"Output: SUBSTANCE: diesel exhaust; SUBSTANCE: rock dust; PLACE: mine;
   ACTIVITY: dust drilling; ACTIVITY: drilling rock; ACTIVITY: loading ore;
   ACTIVITY: loading rock; ACTIVITY: crushing ore; ACTIVITY: crushing rock\n
   "
"\n"
"4. Sentence: The diesel particulate matter was collected on SKC 37mm
   diameter cassettes with QuartzFilter fibre (DPM cassettes).\n"
"Output: SUBSTANCE: diesel particulate matter; DEVICE: SKC 37mm diameter
   cassettes with QuartzFilter fibre; DEVICE: DPM cassettes\n"
```

2.2.4 Six-shots with two examples of DiscNEs (overlapping and non-overlapping)

```
"Examples:\n"
"1. Sentence: Sampling of personal exposure to elemental carbon, nitrogen
   dioxide and inhalable dust was recorded.\n"
"Output: SAMPLE: Sampling of personal exposure; SUBSTANCE: elemental carbon;
    SUBSTANCE: nitrogen dioxide; SUBSTANCE: inhalable dust\n"
"\n"
"2. Sentence: Eligible subjects were identified from the company health-
   service registry according to the following entry criteria: 1) no history
    of respiratory disorder, including bronchitis or any other respiratory
   symptoms.\n"
"Output: NA\n"
"\n"
"3. Sentence: The most important sources include diesel exhaust and rock
   dust, which are vortexed into the air by the traffic in the mine, and/ or
    dust drilling, loading and crushing of the rock/ore.\n"
"Output: SUBSTANCE: diesel exhaust; SUBSTANCE: rock dust; PLACE: mine;
   ACTIVITY: dust drilling; ACTIVITY: drilling rock; ACTIVITY: loading ore;
   ACTIVITY: loading rock; ACTIVITY: crushing ore; ACTIVITY: crushing rock\n
   "
"\n"
"4. Sentence: The diesel particulate matter was collected on SKC 37mm
   diameter cassettes with QuartzFilter fibre (DPM cassettes).\n"
"Output: SUBSTANCE: diesel particulate matter; DEVICE: SKC 37mm diameter
   cassettes with QuartzFilter fibre; DEVICE: DPM cassettes\n"
"\n"
"5. Based on these assumptions, temperature and CO2 for P&D nonsmoking
   drivers (smoking would have artificially inflated CO2 levels in the cab)
   were analyzed.\n"
"Output: SUBSTANCE: CO2; TITLE: P&D drivers; SUBSTANCE: CO2\n"
"\n"
```

```
"6. Sentence: There were no significant differences in particle exposures
   across the two driver groups, with the exception of significantly higher
   OC and PM2.5 concentrations in the subset of nonsmoking LH drivers
   compared with nonsmoking P&D drivers (p < 0.01).\n"
"Output: TITLE: driver; SUBSTANCE: OC; SUBSTANCE: PM2.5; TITLE: LH drivers;
   TITLE: P&D drivers\n"
```
